# Viral genome editing methods and applications in the CRISPR era

**DOI:** 10.1128/jvi.02048-25

**Published:** 2026-02-18

**Authors:** Kihye Shin, Eui Tae Kim

**Affiliations:** 1 Department of Microbiology and Immunology, Jeju National University College of Medicine 37984 , Jeju, Republic of Korea; 2 Jeju Research Center for Natural Medicine, Jeju National University Core Research Institute 34926, Jeju, Republic of Korea; Universiteit Gent, Merelbeke, Belgium

**Keywords:** herpesvirus genetics, homology-directed repair, CRISPR-Cas genome editing

## Abstract

CRISPR-Cas systems have transformed viral genetics by enabling precise and efficient manipulation of large DNA virus genomes. This review provides a practical framework for applying CRISPR technology to herpesviruses and other large DNA viruses as an alternative and complement to traditional BAC recombination. Key considerations include nuclease choice; sgRNA design that minimizes cut-to-edit distance and prevents re-cutting; donor template configuration and homology arm length; and synchronized delivery of Cas complexes and donor DNA. Strategies to promote HDR efficiency, such as the use of small-molecule modulators, are also summarized. In addition, practical workflows for clone selection, genotypic validation, and phenotypic confirmation are summarized. Case studies in herpes simplex virus type 1 and human cytomegalovirus illustrate how optimized CRISPR designs achieve reproducible, scarless knock-ins and conditional gene manipulation at essential loci without complementing cell lines. Together, these approaches establish CRISPR as a flexible, scalable platform for functional genomics, antiviral target discovery, and translational virology, enabling direct editing of clinical isolates previously inaccessible with bacterial artificial chromosome-based methods.

## INTRODUCTION

### Conventional viral gene editing

Large DNA viruses, including poxviruses, adenoviruses, and herpesviruses, have long served as important models and platforms for molecular genetics and vaccine development. Their genomes are typically between 100 and 300 kilobases in size and contain many regulatory regions, which make precise genetic manipulation technically challenging.

Before the advent of programmable nucleases, most viral genome engineering relied on *in vitro* recombination or bacterial artificial chromosome (BAC) cloning systems. These methods enabled targeted modification of several large DNA viruses, such as herpes simplex virus type 1 (HSV-1) and human cytomegalovirus (HCMV), by combining homologous recombination with counter-selection markers. Because of their efficiency and reproducibility, BAC-based strategies became the standard for functional studies across diverse herpesviruses and other large DNA viruses (1–3).

Nevertheless, these methods are labor-intensive, often require complementing cell lines and complex selection procedures and are restricted to strains for which BAC clones have been established. In addition, scarless editing requires extra steps, and residual BAC sequences frequently need to be eliminated to restore the authentic viral genome (4). By contrast, CRISPR-based approaches provide a more direct and versatile platform. Guide RNA and Cas nuclease are sufficient to introduce targeted DNA cleavage within infected cells, allowing precise modification of viral genomes without BAC intermediates. Recent studies have demonstrated that CRISPR can be used to edit the genomes of HSV-1 and HCMV or suppress their replication in infected cells, supporting its potential to meet the growing demands of modern virology and therapeutic development (5–7).

### CRISPR-Cas-mediated genome editing

The development of CRISPR and associated endonucleases has transformed genome editing across cellular organisms and viruses alike (8–12). In CRISPR-Cas systems, a guide RNA hybridizes with the target sequence and directs nucleases such as Cas9, Cas12, or Cas13 to the specified locus, resulting in cleavage and subsequent modification of the target nucleic acid. Cas9 and Cas12 target DNA, whereas Cas13 acts on RNA (13, 14).

While this review focuses primarily on DNA virus genome editing, the RNA-targeting Cas13 platform extends CRISPR capabilities to the transcript level, offering complementary strategies for manipulating viral gene expression through RNA knockdown or base editing. In infection models, Cas13 systems have been applied for modification of both viral and host transcripts, particularly targeting RNA viruses such as influenza (15), SARS-CoV-2 (15), dengue virus (16), and HIV-1 (17). These RNA-level interventions may provide advantages in specific contexts, such as transient modulation of viral gene expression or targeting viral transcripts without permanently altering the genomic DNA template. For readers interested in detailed case studies of Cas13 applications, we recommend recent comprehensive overviews (18–20).

The most widely used nuclease is *Streptococcus pyogenes* Cas9 (SpCas9), which introduces a double-strand break (DSB) at a DNA site defined by a single-guide RNA (sgRNA). In eukaryotic cells, such breaks are mainly repaired by non-homologous end joining (NHEJ) or microhomology-mediated end joining (MMEJ), typically leading to insertions or deletions (indels) and loss-of-function mutations. When a donor template is available, however, homology-directed repair (HDR) can enable precise sequence integration. This dual repair potential allows CRISPR to mediate both gene knockout and precise knock-in, including single-nucleotide substitutions and tag insertions, within the same platform (13, 21–23). Variants that avoid DSB formation, including Cas9 nickase (nCas9), which generates site-specific single-strand nicks, catalytically inactive Cas9 (dCas9) fused to transcriptional effectors for gene repression (CRISPRi) or activation (CRISPRa) (24), and ultracompact nucleases such as CasMINI that enable genome editing with minimal cargo size, further expand system versatility (25–28).

Beyond Cas9, several alternative nucleases have been developed for genome editing. Cas12 family members (Cas12a, Cas12b, and Cas12f) generally recognize T-rich protospacer adjacent motifs (PAMs) and introduce staggered cuts at PAM-distal sites, creating 5′ overhangs (29). These staggered breaks can be advantageous for HDR in certain designs. Because cleavage occurs at a distance from the PAM, early indels often leave the seed region of the protospacer intact, which allows repeated cleavage and progressive deletions through iterative NHEJ cycles until the PAM or protospacer sequence is lost (30). Cas12f, in particular, has been noted for its ability to induce large deletions when paired guides are used, making it suitable for targeted excision strategies (31, 32). The compact Cas12f offers advantages for vector delivery. Recent protein engineering has generated Cas12f variants with substantially improved editing activity in mammalian cells, broader PAM compatibility, and enhanced capacity for paired-guide designs that promote defined deletions (32).

Over the past decade, the application of CRISPR-Cas technology in virology has expanded rapidly. These systems have been applied not only to cellular genomes but also to DNA viruses and integrated DNA intermediates of RNA viruses, supporting diverse purposes including antiviral therapy, functional dissection of viral factors, and engineering of vaccine vectors. The programmable cleavage capacity of CRISPR enables precise modification at defined sites within viral genomes, facilitating gene knockouts, knock-ins, and point mutation corrections with speed and flexibility. Importantly, this approach allows scarless introduction of desired mutations without residual sequences, providing an attractive alternative to BAC-based recombination for the genetic manipulation of herpesviruses and other large DNA viruses.

### DNA viral gene editing using CRISPR-Cas9

Over the past decade, the scope of CRISPR-Cas9 applications has expanded rapidly to include not only cellular genomes but also DNA viruses and even integrated DNA intermediates of RNA viruses (33). Major applications in virology include the development of antiviral strategies, functional studies of viral determinants, and engineering of vaccine vectors (34–37). While the underlying principles are similar to those in host genome editing, viral systems present unique opportunities and challenges. During the lytic phase, viral genomes are replicated in large numbers, increasing the likelihood of editing events. Replication compartments in the nucleus provide localized environments enriched in both viral proteins and host DNA repair factors, creating conditions favorable for CRISPR-mediated editing (38, 39). Moreover, virus-specific phenotypic readouts, such as plaque formation or growth curves, enable rapid functional validation, and clonal viral populations can be readily obtained through plaque purification or endpoint dilution.

At the same time, viral genome editing is sensitive to experimental timing. The infection stage, multiplicity of infection (MOI), and the timing of Cas9 and donor template delivery strongly influence editing outcomes. High copy numbers of viral genomes also increase the risk of repeated cleavage at the same locus, which may lead to progressive large deletions or unintended rearrangements. To minimize these events, donor constructs can be designed with silent mutations in the PAM or seed region to prevent re-cleavage. When essential genes are targeted, compensatory mutations or recombination-driven escape variants may arise rapidly; in such cases, conditional expression systems or cellular complementation provide important safeguards.

Previous reviews of CRISPR use in virology have largely focused on vaccine development, therapeutic applications, diagnostics, and host-virus interactions (34, 40–44). Here, we highlight the following practical considerations specific to viral genome editing.

- Tool selection: choice of nuclease and sgRNA design based on gene essentiality, PAM availability, target site position (frameshift vs. precise edit), and prevention of re-cutting.
- Delivery strategy: optimization of RNPs, plasmids, or viral vectors depending on the infection model.
- Donor design*:* selection of single-stranded oligodeoxynucleotide (ssODN) or double-stranded DNA (dsDNA) templates depending on the intended modification and screening approach.
- Screening and validation: verification of on-target editing by junction PCR or amplicon sequencing; clonal virus isolation by plaque purification or endpoint dilution; genome-wide validation by whole-genome or long-read sequencing to assess deletions or rearrangements; and phenotypic validation by growth kinetics, plaque size, and viral yield. For essential genes, rescue or revertant experiments are recommended to confirm specificity.

CRISPR-based genome editing has now been demonstrated in a variety of DNA viruses, including herpesviruses, polyomaviruses, papillomaviruses, and poxviruses. Reported applications range from targeted knockouts and knock-ins to the generation of vaccine strains. Representative examples are summarized in Table 1.

**TABLE 1:** Representative studies of CRISPR-Cas-mediated editing in viral genomes

Virus	Target gene/modification	Editing strategy	Delivery of CRISPR editing components	Notes/outcome	Reference
HSV-1	gE knockout and revertant	HDR	Co-transfection of Cas9/gRNA and ssODN DNA	First CRISPR editing report in HSV-1	(45)
HSV-1	UL23 with EGFP insertion	HDR	Co-transfection of Cas9/gRNA and donor plasmids	Reporter knock-in	(46)
HSV-1	ICP0 knock-in	HDR	Stable Cas9/gRNA 293T cell; donor plasmid transfection	Precise insertion	(47)
HSV-1	ICP0 knockout	NHEJ	Transfection of Cas9/gRNA plasmid	Loss-of-function mutant	(48)
HSV-1	TdTomato insertion (UL26–UL27)	HDR	Co-transfection of Cas9/gRNA and donor plasmids	Reporter virus	(49)
HSV-1	Genome-wide KO (2D/3D)	NHEJ	Transduction of AAV expressing Cas9/gRNA	Editing validated in 3D culture	(50)
HCMV	US34 knock-in	HDR	Stable Cas9/gRNA MRC5 cell; donor DNA electroporation	Reporter insertion	(5)
HCMV	IE1 knockout	NHEJ	Transduction of lentivirus expressing Cas9/gRNA	Functional dissection	(51)
HCMV	MIEP knockout	NHEJ	Transduction of lentivirus expressing Cas9/gRNA	Promoter deletion	(52)
VZV	ORF63 knockout	NHEJ	Transduction of lentivirus expressing Cas9/gRNA	Latency-associated gene study	(53)
DEV	Vaccine strain modification	NHEJ/HDR	Co-transfection of Cas9/gRNA and donor plasmids	Attenuated vaccine	(54)
Marek’s disease virus (MDV)	V5/GFP tagging	HDR	Electroporation of Cas9/gRNA plasmid and donor DNA	Reporter vaccine strain	(55)
PRV	Knock-in/dual reporter (GFP, Luc)	HDR	Co-transfection of Cas9/gRNA and donor plasmids	Dual-reporter virus	(56, 57)
Turkey herpesvirus	Knockout/tagging	NHEJ	Co-transfection of Cas9/gRNA and donor plasmids	Vaccine vector modification	(58)
EBV	Knockout and knock-in episome	NHEJ/HDR	Co-transfection of Cas9/gRNA and donor plasmids	Functional analysis	(59)
KSHV	ORF57 knockout (BCBL-1)	NHEJ	Transfection of Cas9/gRNA plasmid	Latency gene editing	(60)
HBV	Episomal DNA knockout	NHEJ	Transfection of Cas9/gRNA plasmid	Replication inhibition	(61)
JC polyomavirus	Knockout in 2D/3D models	NHEJ	Transduction of lentivirus expressing Cas9/gRNA	Functional analysis	(62, 63)
HIV-1	Provirus knockout	NHEJ	Transduction of AAV expressing Cas9/gRNA	Targeting integrated genome	(64)
HPV	E7 knockout (plasmid injection)	NHEJ	Nanoparticle-mediated transfection of Cas9/gRNA plasmid	Cervical cancer prevention	(65)
HPV18	E6 knockout (AAV-delivered Cas9)	HDR	Transduction of AAV expressing Cas9/gRNA	HeLa model	(66)
Vaccinia virus (VACV)	Genome editing	NHEJ/HDR	Electroporation of Cas9/gRNA and donor plasmids	Smallpox vaccine vector	(67)
HSV-1	UL36 tagging	HDR	Co-transfection of Cas9/gRNA and donor plasmids	UL36 essential gene two-step KI using IRES	(68)
HCMV	IE1/IE2 tagging	HDR	Co-transduction of Cas9/gRNA and donor IDLVs	Inducible degron system knock-in	(69)

## CONSIDERATION FOR EFFICIENT GENOME EDITING

**Fig 1:**
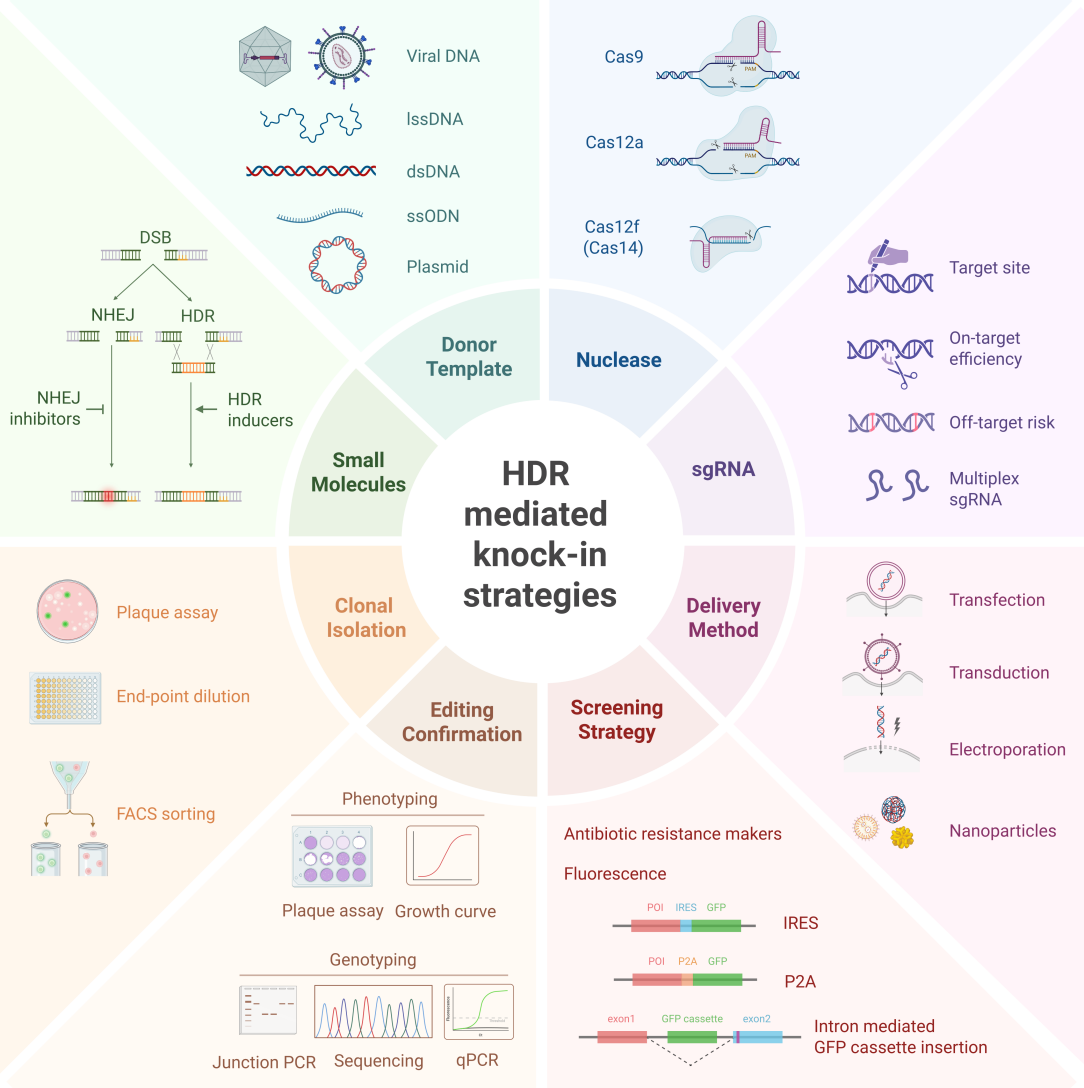
Overview of HDR-mediated knock-in strategies. Schematic summary of key components influencing HDR efficiency, including nuclease selection, sgRNA design, donor template configuration, delivery methods, screening strategies, and clonal validation approaches.

Efficient genome editing ultimately hinges on steering DNA repair toward the desired pathway. Because NHEJ is active across most cellular contexts, gene knockout is typically achievable with relatively simple design decisions, although the resulting indels are stochastic and limit precision. By contrast, HDR-based knock-in affords precise sequence changes but operates within a narrow window of conditions. Performance depends on nuclease properties (PAM, cleavage pattern, and cut position), donor format and length, strand bias, cut-to-edit distance, silent mutations to block re-cutting, delivery method and timing, and, in some settings, small-molecule modulation. In what follows, we emphasize determinants that most strongly influence knock-in outcomes (Fig. 1).

### Selection of CRISPR-Cas proteins

The choice of nuclease should be guided by the editing goal (knockout or knock-in), the availability of PAM sequences near the target locus, and the characteristics of the cut site. SpCas9 is generally the first option, as it is the most widely used nuclease and supported by extensive protocols and validation tools. However, Cas12 family members, particularly compact nucleases such as Cas12f, can serve as useful alternatives when PAM sequences are limited, when multiplex editing is required, or when vector capacity is restricted.

#### Cas9

SpCas9 is a type II nuclease that recognizes its target through an sgRNA and cleaves dsDNA using its HNH and RuvC domains, typically three nucleotides upstream of the NGG PAM (70). Engineered variants such as SpCas9-NG (recognizing NG PAMs) and SpG/SpRY (capable of near-PAM-less targeting) expand the range of accessible target sites (71). To reduce off-target cleavage, high-fidelity variants (eSpCas9 and SpCas9-HF) and paired nicking strategies using nCas9 are effective for precise editing (72, 73). SaCas9 from *Staphylococcus aureus* is smaller and recognizes an alternative PAM (NNGRRT), making it particularly useful when vector packaging capacity is limited or PAM availability is constrained (74).

#### Cas12a (Cpf1)

Cas12a (type V) recognizes T-rich PAM sequences (commonly TTTV) and generates staggered 5′ overhangs at PAM-distal cut sites located approximately 18–23 nucleotides from the PAM. It operates with a crRNA alone and is capable of processing a single RNA transcript into multiple crRNAs, which enables multiplex editing using one promoter (75, 76). Cas12a is advantageous when NGG PAMs are scarce or when simultaneous disruption of multiple loci is required.

#### Cas12f (Cas14)

Cas12f is a small nuclease (~400–600 amino acids) that has shown improved activity in mammalian cells through protein engineering. Its compact size makes it compatible with packaging into adeno-associated virus (AAV) vectors, which is an important consideration for delivery in viral systems. However, PAM preferences differ between Cas12f variants; hence, locus-specific screening and optimization of guide design are usually required (25, 77).

### Selection of sgRNAs

Designing sgRNAs requires balancing on-target activity, off-target risk, and cut placement relative to the desired edit. For knockout, the main goal is to ensure robust cleavage, since even random indels are sufficient to disrupt gene function if they cause frameshifts. For knock-in, however, cleavage must be carefully positioned close to the intended modification site, and donor design must include silent mutations that prevent re-cleavage after HDR.

#### sgRNAs for knockout

A practical workflow begins with *in silico* prediction followed by experimental validation. Web-based prediction tools can prioritize candidates based on expected cleavage efficiency and off-target probability (78–82). These scores are useful for initial filtering, but they are not fully reliable, as performance varies by cell type and locus. Final activity must therefore be confirmed experimentally, for example, by *in vitro* cleavage assays using Cas protein and sgRNA or by amplicon sequencing after transfection (70, 83–85).

When designing sgRNAs for knockout, it is best to target constitutive coding exons shared by all isoforms. Cuts placed within the central portion of such exons increase the likelihood of frameshifts and premature stop codons (70, 80, 86). Guides that cut too far upstream or downstream may allow production of partially functional proteins or the use of alternative start codons. In such cases, targeting near-essential protein domains or using paired sgRNAs to delete critical intervals can improve knockout efficiency.

In rapidly replicating viral systems, editing of essential genes can generate strong growth defects that enrich for escape variants. To avoid misinterpretation, complementation approaches, conditional expression or degradation systems, or the use of multiple sgRNAs to enforce large deletions should be considered.

#### sgRNAs for knock-in

For knock-in, the distance between the cleavage site and the desired edit is critical because HDR efficiency decreases as this distance increases (87). Therefore, the best strategy is to select sgRNAs that cut as close as possible to the intended insertion or substitution site. After HDR, re-cleavage of the edited allele can occur if the PAM or seed region is left intact. This can be avoided by introducing silent mutations into the donor sequence that disrupt the PAM or seed without altering protein coding (36, 75, 88, 89). If this is not possible, alternative guides or nucleases should be considered. Some studies report that using two sgRNAs can increase knock-in efficiency by promoting larger insertions, although this also increases the risk of off-target effects (90).

### Donor templates

Precise knock-in or replacement requires a donor template to be present during the HDR window. Donor type, delivery method, and homology arm design depend on cell type, infection model, and insert size. For the best outcomes, the donor should be available at high nuclear concentration at the time of nuclease activity.

#### Donor formats

- ssODN or long single-stranded DNA (lssDNA): best for small edits such as SNPs or short insertions. Synthesis is straightforward, risk of random integration is low, and asymmetric homology arms can improve HDR efficiency. The main limitation is length, typically ≤200 nt for ssODN in total and a few hundred nt for lssDNA (91–95).
- Linear dsDNA/plasmid dsDNA: suitable for larger insertions (hundreds of bp to several kb). Linear dsDNA integrates quickly but can undergo unwanted end-joining. Plasmid donors are stable but may persist in the nucleus, necessitating clonal validation. *In vivo* linearization, achieved by placing Cas target sites near homology arms of the donor template, can synchronize cleavage of both target and donor, improving recombination through HDR or HMEJ (96, 97).
- AAV: provides high knock-in efficiency across many cell types and can package up to ~4.5 kb. Particularly effective in primary cells and *in vivo* settings but constrained by packaging limits and production costs (98).
- IDLV (integrase-deficient lentivirus vector): support the delivery of larger cassettes with lower integration risk compared to standard lentivirus. However, low-level random integration still occurs, requiring plaque purification and sequencing validation (99).
- Silent mutations: regardless of donor type, silent PAM/seed mutations should be incorporated to prevent re-cutting after HDR.

#### Homology arm length

- ssODN: each homology arm is typically ~30–60 nt (total donor length 100–200 nt), which aligns with the synthesis limit described above.
- lssDNA: ~150–400 nt per arm (total several hundred nt to ~1 kb).
- Linear dsDNA/plasmid: 300–800 bp per arm is common, with longer arms supporting more reliable large insertions.
- Cas9 blunt cuts: knock-in efficiency is highest when edits lie within ±5–10 bp of the cut site.
- Cas12a staggered cuts: the position of the staggered overhang relative to the edit can influence efficiency.

### Delivery methods for CRISPR components

**Fig 2:**
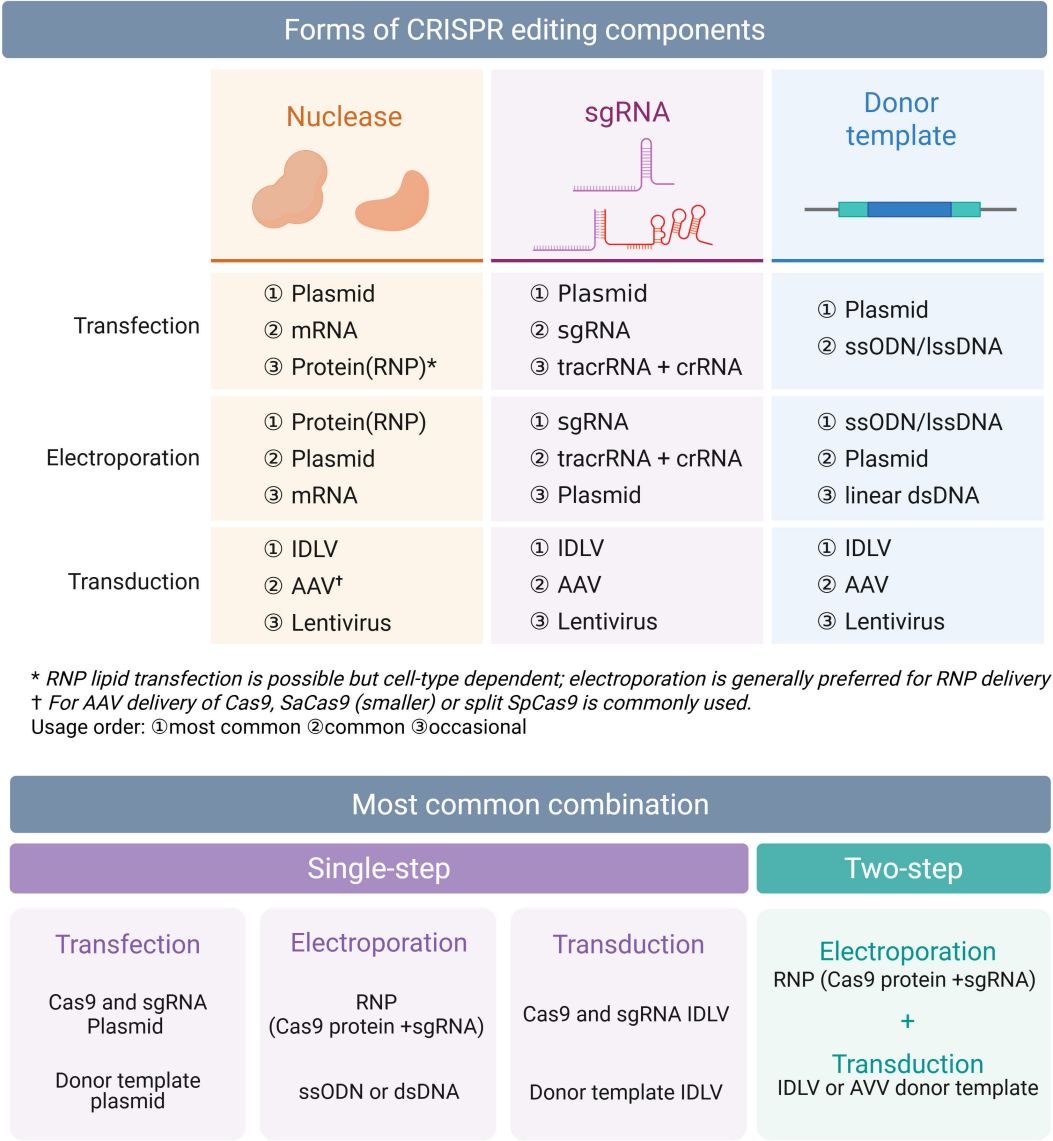
Delivery formats and combinations for CRISPR-Cas-mediated genome editing. Summary of typical forms of Cas nucleases, sgRNAs, and donor templates used for transfection, electroporation, or transduction. Common single-step and two-step combinations are highlighted, illustrating optimal configurations for HDR-based viral genome editing.

In viral genome editing, efficient delivery is often the limiting step. For HDR-based knock-in, Cas nuclease, sgRNA, donor template, and viral genome must coincide within the same cell and nucleus during the HDR window (100–102). Each delivery method also imposes restrictions on the forms of CRISPR components it can accommodate, and efficiency varies with format (Fig. 2).

Recombination is most active shortly after cleavage; hence, the donor should reach peak nuclear concentration at that time. Co-delivery of RNP plus donor by electroporation aligns cut initiation with donor availability and shortens nuclease exposure, which often reduces off-target effects and re-cutting. With plasmid transfection, expression delay can desynchronize cleavage; in this case, donors should be provided concurrently or within 6–12 hours, and in some systems, a second “booster” transfection is beneficial. AAV donors usually reach maximum nuclear levels within several hours to 1 day after infection, while IDLV donors peak later. Thus, MOI and infection timing should be optimized, taking into account the dynamics of viral replication compartments. When IDLV donors are combined with RNP, one effective approach is to perform IDLV transduction first, followed by RNP electroporation about 24 h later (103).

#### Transfection

Lipid-based transfection remains one of the simplest and most reproducible delivery methods. It is particularly effective in permissive cell lines such as HEK293, HeLa, or Vero, where high transfection efficiency allows straightforward proof-of-concept studies. It is also cost-effective and compatible with both plasmid DNA and donor oligonucleotides. However, its performance is strongly cell line-dependent, and efficiency drops in primary or suspension cells. For HDR, one limitation is that plasmid expression involves a lag period, which can cause misalignment between nuclease cleavage and donor availability. To address this, donors should be supplied at the same time or within 6–12 h, and booster transfections may improve overlap. Recently, commercial reagents have been optimized for direct RNP delivery, increasing editing efficiency and reducing off-target effects (104). Overall, transfection is best-suited for initial optimization, but careful attention to timing is required for knock-in applications.

#### Electroporation

Electroporation is the most versatile and efficient method for hard-to-transfect cells, including primary cells, monocytes, CD34+ hematopoietic progenitors, and suspension lines. It is especially effective for delivering Cas9 RNP, which allows immediate cleavage upon entry and rapid nuclease degradation, minimizing off-target accumulation (105–109). Electroporation can co-deliver a wide range of donor types (ssODN, lssDNA, linear dsDNA, and plasmid DNA), making it flexible for both small and large knock-in designs (107–109). However, optimization of pulse conditions, buffer composition, and cell density is essential, as excessive stress can reduce viability. The method also requires specialized instrumentation and can become costly in large-scale experiments. Despite these drawbacks, its combination of high efficiency and compatibility with diverse donor forms makes electroporation the preferred strategy for demanding viral editing contexts.

#### Viral vectors

Viral vectors offer unique advantages when stable or efficient delivery is required. Integrating lentivirus supports long-term Cas9/sgRNA expression, which is useful for screens or systems requiring sustained activity. However, random integration is a concern, especially in therapeutic contexts. IDLV provides transient expression and is widely used for donor delivery. IDLV accommodates larger inserts compared to AAV, and several studies have reported all-in-one IDLV systems where Cas9, sgRNA, and donor are packaged together (99, 110–112). This approach is particularly attractive for editing large viral genomes such as herpesviruses. However, low-level integration events can occur; hence, plaque purification and whole-genome sequencing are recommended for validation. Recombinant AAV (rAAV) has become a gold standard donor vector for precise knock-in. Its ability to deliver single-stranded DNA directly into the nucleus supports high HDR efficiency even in primary cells or *in vivo* (113–115). In herpesvirus genome editing, rAAV donors have been widely used to introduce tags or deletions that are difficult to achieve with plasmid donors. Recent innovations include capsid engineering (e.g., Y704T variants) to enhance nuclear trafficking and capsid-tethered HDR-promoting factors to improve recombination specificity. Nevertheless, rAAV is constrained by a packaging limit of ~4.7 kb, carries a low but detectable risk of random integration, and may provoke immune responses to viral proteins (116–119).

#### Other methods

Several additional methods provide niche advantages. Calcium phosphate transfection remains inexpensive and highly reproducible in HEK293 cells, although it is inefficient and often toxic in primary cells (120, 121). Lipid nanoparticles (LNPs) can encapsulate Cas9 mRNA with sgRNA or Cas9 RNP, offering transient expression with low immunogenicity (122, 123). This makes them particularly useful where electroporation is toxic. Cell-penetrating peptides conjugated to Cas9 RNP have enabled direct delivery into sensitive or neuronal cells and are also being tested for base editing and prime editing (124, 125). Magnetofection, which uses magnetic nanoparticles to concentrate editing components on the cell surface, has achieved strikingly high uptake (>99%) and notable knock-in efficiencies (~43%) in iPSC-derived neural progenitors (126–129). Although still experimental, such approaches highlight the diversity of delivery platforms under exploration.

### Small molecules to enhance HDR

Cells primarily repair DSBs through NHEJ, whereas HDR is restricted to the S and G2 phases of the cell cycle. Upon DSB induction, DNA damage response pathways are rapidly activated (130). In G1 or M phase, end resection is suppressed, and NHEJ dominates, while in S/G2 phase, end resection is activated, promoting HDR (131).

In host genome editing, cell cycle modulators such as nocodazole, vinblastine, and RO-3306 increase HDR by synchronizing cells in G2/M, when homologous recombination is most active (132, 133). Similarly, XL413, a CDC7 inhibitor, prolongs S phase duration, providing an extended time window for HDR (134). However, these strategies may be less effective in viral genome editing, since infection proceeds asynchronously across the cell population, but they can still be tested empirically.

NHEJ is initiated by the Ku70/Ku80 heterodimer binding to DSB ends, preventing further degradation. This recruits DNA-PKcs, which together form the presynaptic complex. Long-range synapsis is first established, followed by XRCC4-XLF recruitment, which bridges the DNA ends and enables ligation by DNA ligase IV (135, 136). In contrast, HDR initiation depends on recruitment of the MRN complex (MRE11-RAD50-NBS1). MRE11 provides Mn²^+^-dependent endonuclease activity, RAD50 forms a homodimer that constitutes the structural core, and NBS1 coordinates downstream repair proteins. These mechanistic differences explain why blocking NHEJ or promoting HDR can substantially influence editing outcomes.

#### NHEJ inhibitors

Several small molecules that inhibit NHEJ have been used to enhance HDR. DNA-PKcs inhibitors such as NU7026 (an ATP-competitive inhibitor) and M3814 (peposertib) increase HDR efficiency by approximately 2-fold to 4-fold in systems such as iPSCs, HEK293, K562, and T cells (115, 137–139). High-throughput screens also identified AZD7648, which produced >50-fold enhancement in some assays (140–142). However, potent DNA-PK inhibition has been linked to large deletions, chromosomal arm loss, and translocations, raising concerns about genomic instability (143). Inhibition of DNA ligase IV by SCR7 prevents ligation of DNA ends, thereby blocking NHEJ, although its reported effects are inconsistent across cell types and target loci. Moreover, SCR7 can show dose-dependent cytotoxicity, necessitating careful titration (131, 138, 144–146).

#### HDR activators

Compounds that directly stimulate homologous recombination have also been explored. RS-1 enhances HDR by stabilizing RAD51 filaments on single-stranded DNA (ssDNA), thereby promoting strand invasion, although the results vary significantly between cell types (138, 147, 148). Chromatin remodeling approaches provide another avenue: histone acetylation relaxes chromatin into a euchromatic state, increasing DNA accessibility. Inhibitors of histone deacetylases (HDACs), such as trichostatin A (TSA), increased HDR up to 4-fold in iPSCs (149). However, HDAC inhibitors are associated with cytotoxicity and apoptosis at higher doses, limiting their general utility. In addition, inhibition of ATR (VE-822) and CHEK1 (AZD-7762) has been reported to alter DNA damage responses in ways that bias repair toward HDR, although their broader application in viral editing has yet to be fully validated (150).

Collectively, these studies demonstrate that HDR efficiency can be modulated by manipulating either the cell cycle state, blocking NHEJ, or directly stimulating HDR pathways. Representative compounds, their mechanisms, and reported outcomes are summarized in Table 2.

**TABLE 2:** Small-molecule modulators of DNA repair pathways for enhancing HDR efficiency

Category	Compounds	Mechanism	Reported effects	Notes	References
Cell cycle modulators	Nocodazole, Vinblastine, RO-3306	Arrest cells in G2/M phase, favoring HDR	Increased HDR frequency	May be less effective in viral editing due to asynchronous infection	(132, 133)
	XL413 (CDC7 inhibitor)	Extends S phase duration	Provides longer time window for HDR	Effect is system-dependent	(134)
NHEJ inhibitors	NU7026 (DNA-PKcs inhibitor)	Blocks DNA-PKcs kinase activity	2-3 fold increase in HDR (iPSCs, HEK293, K562)	Moderate enhancement, widely used	(137, 138)
	M3814 (Peposertib)	Inhibits DNA-PKcs activity	Increased HDR in iPSCs and T cells	In clinical testing	(139)
	AZD7648	Potent DNA-PKcs inhibitor	>50 fold enhancement in screens	Risk of large deletions, translocations	(140–143)
	SCR7 (Ligase IV inhibitor)	Prevents DNA end ligation by LIG4	Reported suppression of NHEJ	Variable results, possible cytotoxicity	(131, 138, 144–146)
HDR activators	RS-1	Stabilizes RAD51 nucleoprotein filaments	Promotes strand invasion and HDR	Variable efficacy across cell types	(138, 147, 148)
	HDAC inhibitors (e.g., TSA)	Increase chromatin accessibility via histone acetylation	Up to 4-fold increase in HDR (iPSCs)	Cytotoxic at higher doses	(149, 150)
	ATR inhibitors (VE-822)	Block ATR-mediated DDR signaling	Shift repair balance toward HDR	Limited validation in viral editing	(150)
	CHEK1 inhibitors (AZD-7762)	Inhibit CHK1 checkpoint kinase	Promote HDR by altering DNA damage response	Under investigation, potential toxicity	(150)

## SELECTION AND SCREENING METHODS

### Fluorescence-based selection markers

**Fig 3:**
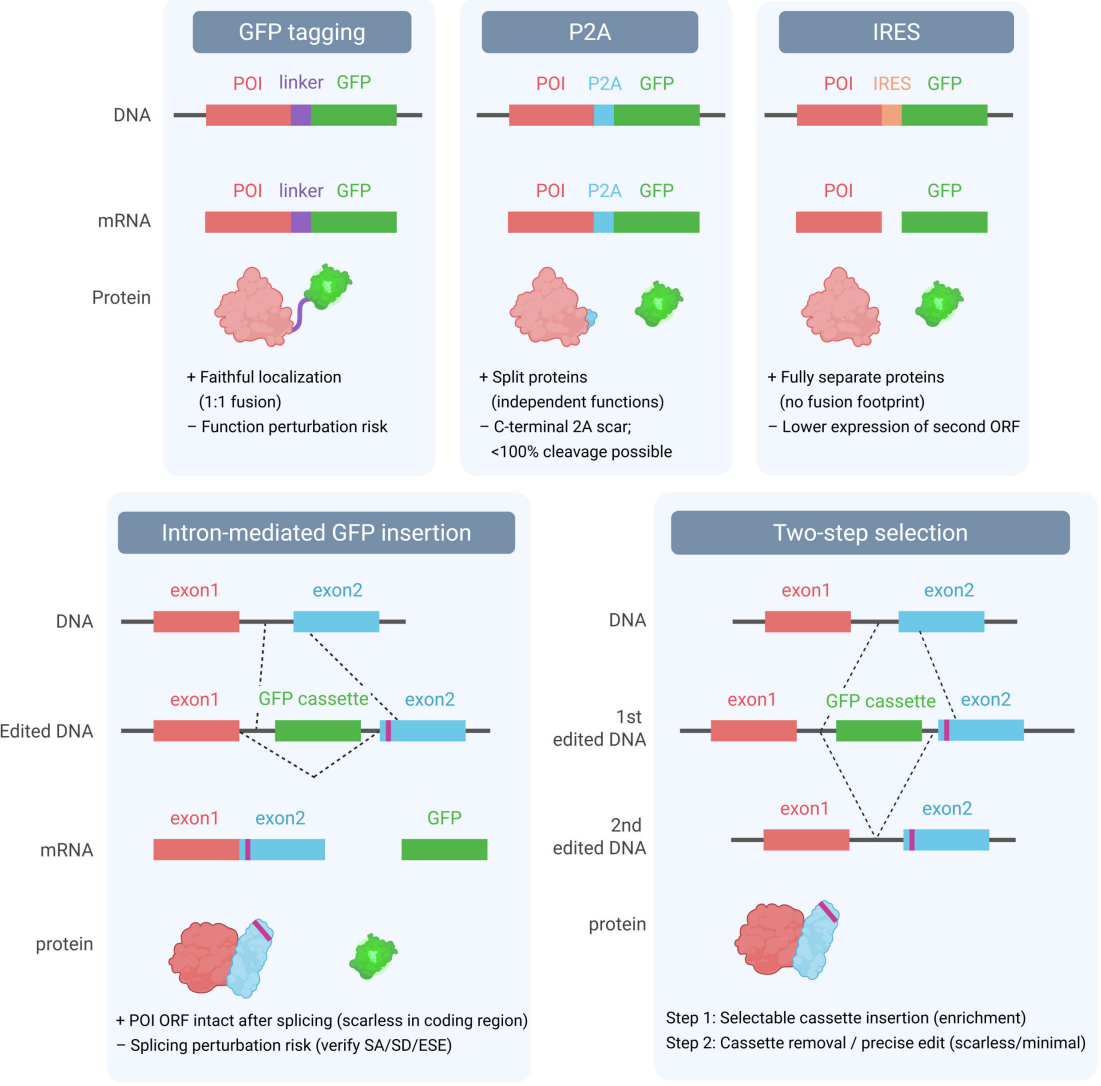
Representative strategies for fluorescence-based selection in viral genome editing. Comparison of commonly used reporter integration approaches, including direct GFP tagging, P2A or IRES bicistronic expression, intron-mediated GFP insertion, and two-step selection systems for scarless editing.

Fluorescent reporters are commonly integrated as markers to enrich or identify successfully edited viral genomes. Several strategies have been developed, each with distinct advantages and limitations (Fig. 3).

#### Internal ribosome entry site (IRES)

IRES elements allow independent translation of two ORFs from a single transcript, thereby preserving the amino acid sequence of the target protein (151, 152). However, translation efficiency is biased toward the upstream ORF, and expression from the downstream ORF is often substantially lower. Thus, a configuration such as GFP-IRES-target gene can compromise expression of the target protein, whereas target gene-IRES-GFP may produce a weak GFP signal and reduce the sensitivity of selection (151, 152).

#### 2A self-cleaving peptides (P2A)

2A peptides, including P2A, enable the production of two nearly equimolar proteins from a single ORF via ribosomal skipping (153). This balanced expression favors reliable selection. The drawback is that 2A sequences leave short peptide scars: additional residues at the C-terminus of the upstream protein and an N-terminal proline on the downstream protein. These extra amino acids can, in some cases, affect protein stability or function (154).

#### Intron-based insertion strategies

Reporter integration within introns provides an alternative, scarless tagging approach. In the GEIS (gene editing through intron-mediated scarless integration) system, CRISPR induces a cut within an intron adjacent to the target exon, and a fluorescent reporter cassette with its own promoter is inserted by HDR (155). This allows visualization of target gene expression without modifying the coding sequence. Since dsDNA donors frequently lead to random integration or spurious expression, single-stranded DNA donors are preferred. These are often generated by heat-denaturing PCR products, and homology arms of at least ~500 nt are typically required for efficient integration.

Another method, CRISPIE (CRISPR-mediated intron-encoded tagging), inserts a synthetic exon module into an intron (155). Upon splicing, the artificial exon becomes part of the mature mRNA, placing the fluorescent tag in-frame with the endogenous coding sequence. This design enables functional tagging of proteins at either terminus while preserving the surrounding genomic context, offering a flexible and scarless way to monitor protein expression (155).

#### Two-step selection

When completely scarless editing is required, a two-step strategy can be applied. In the first step, a removable GFP or drug-resistance cassette is inserted by HDR near the target locus to allow the easy selection of positive clones. In the second step, the cassette is precisely removed, leaving only the intended mutation without any additional sequences (156). Although this approach requires two rounds of editing and increases the risk of off-target events, it ultimately provides the advantage of producing fully scarless genomes, which should be validated by whole-genome sequencing.

Beyond fluorescence-based selection, combined positive/negative systems have also been introduced to improve precision. One example is the CD/UPRT-T2A-NPTII cassette, which links neomycin phosphotransferase II (NPTII) for positive selection with cytosine deaminase/uracil phosphoribosyltransferase (CD/UPRT) for negative selection, separated by a T2A sequence to ensure co-expression. This design allows the first round of selection through antibiotic resistance and the subsequent removal of unwanted clones by drug-induced killing, thereby enriching for scarless edited genomes (157).

### Clonal purification of edited viruses

After genome editing, the initial viral population is heterogeneous, with both edited and unedited genomes often co-existing in the same infected cell. Clonal purification is therefore essential. Moreover, plaque morphology (e.g., size, growth rate) can bias clone selection and carries a risk of inadvertently enriching recombinants or escape variants. Rigorous purification and quality control are therefore mandatory, typically involving two or more rounds of isolation.

#### Plaque purification

Plaque isolation on semisolid overlays (agarose or methylcellulose) remains the standard for DNA viruses (158). Fluorescent reporters can accelerate the process by enabling direct selection of positive plaques. Typically, at least two rounds of plaque purification are required to ensure clonality. Overlay composition influences plaque size and number; hence, the conditions must be optimized for each virus species (159).

#### Cell sorting

When a fluorescent reporter or other detectable marker is available, infected cells can be pre-enriched using flow cytometry. Positive cells are sorted and subsequently subjected to plaque assay or limiting dilution to obtain a clonal virus (160). Reporter-linked editing enables higher clonal recovery rates and shorter timelines. In viruses with weak plaque formation, combining FACS pre-enrichment with end-point dilution is advantageous.

#### End-point dilution

Sequential dilution ensures that individual wells receive, on average, fewer than one infectious unit. Wells showing cytopathic effect are then expanded. This approach is especially useful for viruses that do not form clear plaques or when overlays are not feasible. Viral titers are quantified in parallel by TCID₅₀, using methods such as Reed-Muench or Spearman-Kärber (161, 162).

#### Droplet microfluidics

Microfluidic droplet systems encapsulate single infected cells or virions in nanoliter compartments, enabling high-throughput isolation and analysis (163, 164). Although not yet widely applied to herpesviruses, this approach has been successfully demonstrated with other viruses and may provide future avenues for automated single-virus purification.

#### Selecting appropriate viral purification strategies

For viruses with robust plaque formation (e.g., HSV-1), plaque purification combined with fluorescent reporters is the most efficient. For viruses with weak plaque formation, overlay difficulties, or those requiring primary cells, FACS pre-enrichment followed by end-point dilution is preferred. For high-throughput or rapid processing, droplet microfluidics, potentially combined with AI-driven automation, represents a promising option.

### Confirmation of genome editing

After obtaining isolated candidate clones, both genotypic and phenotypic verification steps are required. The main objectives are as follows: (i) to confirm whether the intended on-target modification has occurred, (ii) to exclude unwanted rearrangements, large deletions, or donor backbone retention (off-target events), and (iii) to assess whether the edited virus maintains expected growth and functional properties.

#### Genotypic assays

On-target editing is commonly verified by junction PCR and Sanger sequencing across the donor-genome junctions to ensure precise recombination. If the design is scarless, the absence of any residual selection cassette should also be confirmed. For quantitative assessment, amplicon-based next-generation sequencing (NGS) can be performed to profile mutation spectra, including HDR efficiency, indel frequency, and frameshift distribution. Mixed sequencing signals indicate heterogeneity within the population and typically warrant additional purification rounds (165).

To evaluate potential off-target effects, the top predicted off-target sites can be amplified and sequenced using targeted amplicon NGS. Depending on the project scale and risk level, long-read sequencing or even whole-genome sequencing may be implemented to identify large deletions, rearrangements, or cryptic integrations that may not be detected by short-read approaches (165).

#### Phenotypic assays

Phenotypic validation is essential to determine whether the edited virus retains expected replication kinetics and protein function. Standard assays include single-step and multi-step growth curves, plaque size comparison, and viral yield quantification relative to wild-type controls. Changes in viral replication may indicate functional alteration of the target gene. Protein expression and integrity can be analyzed by immunoblotting or immunofluorescence using antibodies specific to the target. When a fluorescent or reporter tag is used, its expression can serve as a direct visual confirmation of successful knock-in or inducible system performance.

## APPLICATIONS AND REPRESENTATIVE STUDIES

CRISPR-based genome editing has expanded the experimental and translational scope of herpesvirus research by enabling precise manipulation of large DNA genomes. This technology facilitates functional genomics studies, such as mapping gene essentiality, dissecting protein domains, and probing virus-host interactions, while also advancing applications in vaccine development, antiviral screening, and oncolytic virotherapy. Because herpesviruses possess large genomes with complex regulatory elements, the programmable and modular nature of CRISPR tools makes them particularly suitable for targeted manipulations without global genomic disruption.

### Auxin-inducible degron-mediated protein control in HCMV

Editing HCMV in hard-to-transfect cells such as human foreskin fibroblasts (HFFs) poses a major technical challenge. In this study, an IDLV was used to deliver Cas9/sgRNA together with the donor template into infected cells. The donor template was designed with additional Cas9 cut sites at both ends to promote *in vivo* linearization, thereby enhancing HDR efficiency through improved accessibility and co-localization of donor and target sequences.

An auxin-inducible degron (AID) tag was inserted immediately downstream of the IE1 gene together with a P2A-GFP module, creating a dual-function construct for both visual selection and inducible degradation. In HFF-TIR1 cells, treatment with indole-3-acetic acid (IAA) triggered rapid degradation of the AID-tagged IE1 protein, confirming the feasibility of conditional protein depletion within the viral context. This work established a practical pipeline for applying protein-targeted degradation systems to HCMV without the need for BAC-based recombination, providing a model for posttranslational control of essential viral proteins (69).

### CRISPR-based editing strategy for essential genes in HSV-1

Editing essential viral genes requires strategies that maintain viral viability throughout the process. In this example, a two-step knock-in/knock-replacement method was employed to generate a point mutation in the essential UL36 gene of HSV-1.

First, a CMV promoter-eGFP-IRES cassette was inserted upstream of UL36 using HDR, enabling simultaneous expression of eGFP and the essential UL36 protein. This ensured viral propagation even during intermediate editing stages. Cas9 and sgRNA targeting the UL36 N-terminus were delivered along with a donor plasmid containing ~500 bp homology arms. Following transfection into HEK293 cells and infection at MOI 0.5, progeny viruses were passaged with re-targeting sgRNAs to selectively enrich edited genomes.

From this intermediate CMVp-IRES-GFP-UL36 virus, a second round of HDR was performed to remove the GFP cassette and introduce the C40S point mutation, which abolishes the N-terminal DUB activity of UL36. GFP-negative plaques were selected and validated by sequencing.

This strategy offers several advantages. It preserves essential gene expression during editing, eliminates the need for complementing cell lines, reduces time compared with BAC recombination, and enables scarless editing through cassette replacement. The dual-sgRNA design minimizes NHEJ-mediated artifacts, increasing HDR precision. Moreover, the intermediate GFP-UL36 construct can serve as a versatile platform for generating additional UL36 N-terminal modifications (68).

## CONCLUSION

This review provides a practical framework for applying CRISPR-Cas systems to DNA viral genome editing, demonstrating their potential as both an alternative and a complementary approach to conventional BAC recombination. The focus was to offer an experiment-oriented guide for improving the precision and efficiency of knockout and knock-in strategies in large DNA viruses. We summarized key design considerations, including the choice of Cas nucleases, optimization of sgRNA design to minimize cut-to-edit distance and prevent re-cutting through silent mutations, donor template configuration and homology arm length, co-delivery timing of Cas complexes and donor DNA, and the use of small molecules to promote HDR. In addition, this review outlined practical approaches for clone selection and validation, encompassing both genotypic and phenotypic assays, to ensure accuracy and reproducibility in viral genome editing.

Two recent case studies illustrate how these design principles can be successfully implemented. In one example, an IRES-based two-step editing strategy was employed to introduce a precise C40S point mutation into the essential HSV-1 UL36 gene while maintaining its expression throughout the editing process. This approach enabled accurate modification of an essential gene without the need for a complementing cell line (68). In another case, an IDLV delivery system was used to introduce Cas9, sgRNA, and donor templates into hard-to-transfect human fibroblasts, resulting in efficient knock-in of an AID-P2A-GFP tag downstream of the HCMV IE1 and IE2 loci. Incorporating Cas9 cut sites at both donor ends and optimizing the Cas-to-donor ratio significantly improved HDR efficiency and reproducibility (69). Together, these examples demonstrate how systematically optimized CRISPR workflows can achieve rapid, precise, and scarless genome editing in large DNA viruses without relying on BAC-based systems. Such developments are expected to accelerate functional genetic studies of clinical strains, elucidate mechanisms of viral pathogenesis, and identify novel therapeutic targets.

## PERSPECTIVES

Practical challenges in viral genome editing arise from the intricate relationship between biological constraints and experimental limitations. Editing essential genes often results in nonviable or growth-defective viruses or leads to the rapid selection of adaptive escape mutants. To address this, strategies such as conditional expression or degradation systems, complementing cell lines, and two-step editing designs should be considered at the planning stage to maintain replication competence during genome manipulation.

In the lytic phase, viral genomes replicate multiple copies within nuclear replication compartments, which not only increases opportunities for editing but also raises the risk of cumulative repair events, including large deletions and structural rearrangements. Therefore, careful validation at the junction level is indispensable, and the use of long-read or whole-genome sequencing is strongly recommended before confirming clonal candidates. Editing efficiency is further influenced by cell type, infection stage, MOI, and the timing of Cas nuclease and donor introduction. Because efficient co-delivery of both viral genomes and CRISPR components is technically demanding, optimization must be tailored to each virus-host system.

Although inhibition of the NHEJ pathway can promote HDR, it also introduces the risk of extensive deletions or chromosomal instability. Thus, the use of DNA repair inhibitors requires precise dose optimization and post-editing genome integrity checks. Furthermore, predictive models for sgRNA activity and PAM distribution remain limited for viral genomes, underscoring the need for improved computational tools specialized for viral editing contexts.

Despite these challenges, the unique advantage of CRISPR technology lies in its ability to directly edit clinical isolates without the need for BAC cloning. This capability dramatically enhances experimental speed, accessibility, and adaptability, paving the way for a new era of functional virology. However, CRISPR editing also introduces its own sources of variability, including off-target cleavage, random integration, and background mutations accumulated during viral passaging. Even comprehensive whole-genome sequencing cannot always distinguish these events from naturally occurring mutations.

The most reliable solution is to adopt a revertant-based validation framework that rigorously establishes causality between genotype and phenotype. This includes confirming phenotypic reproducibility across independently edited clones, restoring wild-type function through reversion or complementation, validating on-target junctions and copy numbers by long-read or droplet digital PCR analysis, and employing appropriate negative controls such as scrambled sgRNAs, catalytically inactive Cas variants, or mock-passaged samples. Transparent disclosure of viral passage history and sequencing data will further enhance reproducibility and community confidence.

By integrating these validation standards with the flexibility of CRISPR-based tools, researchers can safely leverage the strengths of the technology, including direct editing of clinical strains, rapid functional interrogation, and scalable design, while maintaining scientific rigor. The continued refinement of CRISPR systems, combined with advances in delivery, bioinformatics, and synthetic virology, will ultimately transform how we manipulate and understand large DNA viruses at the genomic level.

## References

[1] Post LE, Roizman B. 1981. A generalized technique for deletion of specific genes in large genomes: alpha gene 22 of herpes simplex virus 1 is not essential for growth. Cell 25:227–232. doi:10.1016/0092-8674(81)90247-6 6268303

[2] Domi A, Moss B. 2002. Cloning the vaccinia virus genome as a bacterial artificial chromosome in Escherichia coli and recovery of infectious virus in mammalian cells. Proc Natl Acad Sci USA 99:12415–12420. doi:10.1073/pnas.192420599 12196634 PMC129459

[3] Messerle M, Crnkovic I, Hammerschmidt W, Ziegler H, Koszinowski UH. 1997. Cloning and mutagenesis of a herpesvirus genome as an infectious bacterial artificial chromosome. Proc Natl Acad Sci USA 94:14759–14763. doi:10.1073/pnas.94.26.14759 9405686 PMC25110

[4] Tischer BK, Smith GA, Osterrieder N. 2010. En passant mutagenesis: a two step markerless red recombination system, p 421–430. *In* Braman J (ed), In vitro mutagenesis protocols. Vol. 634. Humana Press, Totowa, NJ. 10.1007/978-1-60761-652-8_30 20677001

[5] King MW, Munger J. 2019. Editing the human cytomegalovirus genome with the CRISPR/Cas9 system. Virology (Auckl) 529:186–194. doi:10.1016/j.virol.2019.01.021 PMC6382551 30716580

[6] Amrani N, Luk K, Singh P, Shipley M, Isik M, Donadoni M, Bellizzi A, Khalili K, Sariyer IK, Neumann D, Gordon J, Ruan G-X. 2024. CRISPR-Cas9-mediated genome editing delivered by a single AAV9 vector inhibits HSV-1 reactivation in a latent rabbit keratitis model. Mol Ther Methods Clin Dev 32:101303. doi:10.1016/j.omtm.2024.101303 39610766 PMC11602521

[7] Ying M, Wang H, Liu T, Han Z, Lin K, Shi Q, Zheng N, Ye T, Gong H, Xu F. 2023. CLEAR strategy inhibited HSV proliferation using viral vectors delivered CRISPR-Cas9. Pathogens 12:814. doi:10.3390/pathogens12060814 37375504 PMC10302303

[8] Gwon LW, Badon IW, Lee Y, Kim H-J, Lee SH. 2025. Advances in large-scale DNA engineering with the CRISPR system. Exp Mol Med 57:1902–1912. doi:10.1038/s12276-025-01530-0 40887498 PMC12508223

[9] Zhang M-L, Li H-B, Jin Y. 2024. Application and perspective of CRISPR/Cas9 genome editing technology in human diseases modeling and gene therapy. Front Genet 15:1364742. doi:10.3389/fgene.2024.1364742 38666293 PMC11043577

[10] Pacesa M, Pelea O, Jinek M. 2024. Past, present, and future of CRISPR genome editing technologies. Cell 187:1076–1100. doi:10.1016/j.cell.2024.01.042 38428389

[11] Ansori AN, Antonius Y, Susilo RJ, Hayaza S, Kharisma VD, Parikesit AA, Zainul R, Jakhmola V, Saklani T, Rebezov M, Ullah ME, Maksimiuk N, Derkho M, Burkov P. 2023. Application of CRISPR-Cas9 genome editing technology in various fields: a review. Narra J 3:e184. doi:10.52225/narra.v3i2.184 38450259 PMC10916045

[12] Wang H, La Russa M, Qi LS. 2016. CRISPR/Cas9 in genome editing and beyond. Annu Rev Biochem 85:227–264. doi:10.1146/annurev-biochem-060815-014607 27145843 PMC13384723

[13] Hsu PD, Lander ES, Zhang F. 2014. Development and applications of CRISPR-Cas9 for genome engineering. Cell 157:1262–1278. doi:10.1016/j.cell.2014.05.010 24906146 PMC4343198

[14] Abudayyeh OO, Gootenberg JS, Essletzbichler P, Han S, Joung J, Belanto JJ, Verdine V, Cox DBT, Kellner MJ, Regev A, Lander ES, Voytas DF, Ting AY, Zhang F. 2017. RNA targeting with CRISPR-Cas13. Nature 550:280–284. doi:10.1038/nature24049 28976959 PMC5706658

[15] Blanchard EL, Vanover D, Bawage SS, Tiwari PM, Rotolo L, Beyersdorf J, Peck HE, Bruno NC, Hincapie R, Michel F, Murray J, Sadhwani H, Vanderheyden B, Finn MG, Brinton MA, Lafontaine ER, Hogan RJ, Zurla C, Santangelo PJ. 2021. Treatment of influenza and SARS-CoV-2 infections via mRNA-encoded Cas13a in rodents. Nat Biotechnol 39:717–726. doi:10.1038/s41587-021-00822-w 33536629

[16] Basu M, Zurla C, Auroni TT, Vanover D, Chaves LCS, Sadhwani H, Pathak H, Basu R, Beyersdorf JP, Amuda OO, Elsharkawy A, Mosur V, Arthur RA, Claussen H, Sasser LE, Wroe JA, Peck HE, Kumar M, Brinton MA, Santangelo PJ. 2024. mRNA-encoded Cas13 can be used to treat dengue infections in mice. Nat Microbiol 9:2160–2172. doi:10.1038/s41564-024-01726-6 38839984

[17] Yin L, Zhao F, Sun H, Wang Z, Huang Y, Zhu W, Xu F, Mei S, Liu X, Zhang D, Wei L, Cen S, Hu S, Liang C, Guo F. 2020. CRISPR-Cas13a inhibits HIV-1 infection. Mol Ther Nucleic Acids 21:147–155. doi:10.1016/j.omtn.2020.05.030 32585623 PMC7321785

[18] Zhu G, Zhou X, Wen M, Qiao J, Li G, Yao Y. 2024. CRISPR-Cas13: pioneering RNA editing for nucleic acid therapeutics. Biodes Res 6:0041. doi:10.34133/bdr.0041 39228750 PMC11371277

[19] Shi P, Wu X. 2024. Programmable RNA targeting with CRISPR-Cas13. RNA Biol 21:575–583. doi:10.1080/15476286.2024.2351657 PMC11110701 38764173

[20] Yang H, Patel DJ. 2024. Structures, mechanisms and applications of RNA-centric CRISPR-Cas13. Nat Chem Biol 20:673–688. doi:10.1038/s41589-024-01593-6 38702571 PMC11375968

[21] Cong L, Ran FA, Cox D, Lin S, Barretto R, Habib N, Hsu PD, Wu X, Jiang W, Marraffini LA, Zhang F. 2013. Multiplex genome engineering using CRISPR/Cas systems. Science 339:819–823. doi:10.1126/science.1231143 23287718 PMC3795411

[22] Mali P, Yang L, Esvelt KM, Aach J, Guell M, DiCarlo JE, Norville JE, Church GM. 2013. RNA-guided human genome engineering via Cas9. Science 339:823–826. doi:10.1126/science.1232033 23287722 PMC3712628

[23] Chang HHY, Pannunzio NR, Adachi N, Lieber MR. 2017. Non-homologous DNA end joining and alternative pathways to double-strand break repair. Nat Rev Mol Cell Biol 18:495–506. doi:10.1038/nrm.2017.48 28512351 PMC7062608

[24] Bendixen L, Jensen TI, Bak RO. 2023. CRISPR-Cas-mediated transcriptional modulation: the therapeutic promises of CRISPRa and CRISPRi. Mol Ther 31:1920–1937. doi:10.1016/j.ymthe.2023.03.024 36964659 PMC10362391

[25] Xu X, Chemparathy A, Zeng L, Kempton HR, Shang S, Nakamura M, Qi LS. 2021. Engineered miniature CRISPR-Cas system for mammalian genome regulation and editing. Mol Cell 81:4333–4345. doi:10.1016/j.molcel.2021.08.008 34480847

[26] Ran FA, Hsu PD, Lin C-Y, Gootenberg JS, Konermann S, Trevino AE, Scott DA, Inoue A, Matoba S, Zhang Y, Zhang F. 2013. Double nicking by RNA-guided CRISPR Cas9 for enhanced genome editing specificity. Cell 154:1380–1389. doi:10.1016/j.cell.2013.08.021 23992846 PMC3856256

[27] Qi LS, Larson MH, Gilbert LA, Doudna JA, Weissman JS, Arkin AP, Lim WA. 2013. Repurposing CRISPR as an RNA-guided platform for sequence-specific control of gene expression. Cell 152:1173–1183. doi:10.1016/j.cell.2013.02.022 23452860 PMC3664290

[28] Gilbert LA, Larson MH, Morsut L, Liu Z, Brar GA, Torres SE, Stern-Ginossar N, Brandman O, Whitehead EH, Doudna JA, Lim WA, Weissman JS, Qi LS. 2013. CRISPR-mediated modular RNA-guided regulation of transcription in eukaryotes. Cell 154:442–451. doi:10.1016/j.cell.2013.06.044 23849981 PMC3770145

[29] Karvelis T, Bigelyte G, Young JK, Hou Z, Zedaveinyte R, Budre K, Paulraj S, Djukanovic V, Gasior S, Silanskas A, Venclovas Č, Siksnys V. 2020. PAM recognition by miniature CRISPR-Cas12f nucleases triggers programmable double-stranded DNA target cleavage. Nucleic Acids Res 48:5016–5023. doi:10.1093/nar/gkaa208 32246713 PMC7229846

[30] Khan S, Sallard E. 2023. Current and prospective applications of CRISPR-Cas12a in pluricellular organisms. Mol Biotechnol 65:196–205. doi:10.1007/s12033-022-00538-5 35939208 PMC9841005

[31] Fan M, Bao Y, Berkhout B, Herrera-Carrillo E. 2023. CRISPR-Cas12b enables a highly efficient attack on HIV proviral DNA in T cell cultures. Biomed Pharmacother 165:115046. doi:10.1016/j.biopha.2023.115046 37379644 PMC11228593

[32] Kim DY, Lee JM, Moon SB, Chin HJ, Park S, Lim Y, Kim D, Koo T, Ko J-H, Kim Y-S. 2022. Efficient CRISPR editing with a hypercompact Cas12f1 and engineered guide RNAs delivered by adeno-associated virus. Nat Biotechnol 40:94–102. doi:10.1038/s41587-021-01009-z 34475560 PMC8763643

[33] Panfil AR, London JA, Green PL, Yoder KE. 2018. CRISPR/Cas9 genome editing to disable the latent HIV-1 provirus. Front Microbiol 9:2018. doi:10.3389/fmicb.2018.03107 30619186 PMC6302043

[34] Teng M, Yao Y, Nair V, Luo J. 2021. Latest advances of virology research using crispr/cas9-based gene-editing technology and its application to vaccine development. Viruses 13:779. doi:10.3390/v13050779 33924851 PMC8146441

[35] Yuan M, Zhang W, Wang J, Al Yaghchi C, Ahmed J, Chard L, Lemoine NR, Wang Y. 2015. Efficiently editing the vaccinia virus genome by using the CRISPR-Cas9 system. J Virol 89:5176–5179. doi:10.1128/JVI.00339-15 25741005 PMC4403460

[36] van Diemen FR, Kruse EM, Hooykaas MJG, Bruggeling CE, Schürch AC, van Ham PM, Imhof SM, Nijhuis M, Wiertz EJHJ, Lebbink RJ. 2016. CRISPR/Cas9-mediated genome editing of herpesviruses limits productive and latent infections. PLoS Pathog 12:e1005701. doi:10.1371/journal.ppat.1005701 27362483 PMC4928872

[37] Baddeley HJE, Isalan M. 2021. The application of CRISPR/Cas systems for antiviral therapy. Front Genome Ed 3:745559. doi:10.3389/fgeed.2021.745559 34723245 PMC8549726

[38] Caragliano E, Bonazza S, Frascaroli G, Tang J, Soh TK, Grünewald K, Bosse JB, Brune W. 2022. Human cytomegalovirus forms phase-separated compartments at viral genomes to facilitate viral replication. Cell Rep 38:110469. doi:10.1016/j.celrep.2022.110469 35263605 PMC8924372

[39] Wilkinson DE, Weller SK. 2004. Recruitment of cellular recombination and repair proteins to sites of herpes simplex virus type 1 DNA replication is dependent on the composition of viral proteins within prereplicative sites and correlates with the induction of the DNA damage response. J Virol 78:4783–4796. doi:10.1128/jvi.78.9.4783-4796.2004 15078960 PMC387708

[40] Tang N, Zhang Y, Shen Z, Yao Y, Nair V. 2021. Application of CRISPR-Cas9 editing for virus engineering and the development of recombinant viral vaccines. CRISPR J 4:477–490. doi:10.1089/crispr.2021.0017 34406035

[41] Zhang Y, Li M. 2021. Genome editing technologies as cellular defense against viral pathogens. Front Cell Dev Biol 9:9. doi:10.3389/fcell.2021.716344 PMC8320169 34336867

[42] Najafi S, Tan SC, Aghamiri S, Raee P, Ebrahimi Z, Jahromi ZK, Rahmati Y, Sadri Nahand J, Piroozmand A, Jajarmi V, Mirzaei H. 2022. Therapeutic potentials of CRISPR-Cas genome editing technology in human viral infections. Biomed Pharmacother 148:112743. doi:10.1016/j.biopha.2022.112743 35228065 PMC8872819

[43] Zahedipour F, Zahedipour F, Zamani P, Jaafari MR, Sahebkar A. 2024. Harnessing CRISPR technology for viral therapeutics and vaccines: from preclinical studies to clinical applications. Virus Res 341:199314. doi:10.1016/j.virusres.2024.199314 38211734 PMC10825633

[44] Yang F, Aliyari S, Zhu Z, Zheng H, Cheng G, Zhang S. 2025. CRISPR-Cas: a game-changer in vaccine development and the fight against viral infections. Trends Microbiol 33:650–664. doi:10.1016/j.tim.2025.02.006 40069074

[45] Suenaga T, Kohyama M, Hirayasu K, Arase H. 2014. Engineering large viral DNA genomes using the CRISPR-Cas9 system. Microbiol Immunol 58:513–522. doi:10.1111/1348-0421.12180 25040500 PMC7168497

[46] Bi Y, Sun L, Gao D, Ding C, Li Z, Li Y, Cun W, Li Q. 2014. High-efficiency targeted editing of large viral genomes by RNA-guided nucleases. PLoS Pathog 10:e1004090. doi:10.1371/journal.ppat.1004090 24788700 PMC4006927

[47] Lin C, Li H, Hao M, Xiong D, Luo Y, Huang C, Yuan Q, Zhang J, Xia N. 2016. Increasing the efficiency of CRISPR/Cas9-mediated precise genome editing of HSV-1 virus in human cells. Sci Rep 6:34531. doi:10.1038/srep34531 27713537 PMC5054376

[48] Roehm PC, Shekarabi M, Wollebo HS, Bellizzi A, He L, Salkind J, Khalili K. 2016. Inhibition of HSV-1 replication by gene editing strategy. Sci Rep 6:23146. doi:10.1038/srep23146 27064617 PMC4827394

[49] Russell TA, Stefanovic T, Tscharke DC. 2015. Engineering herpes simplex viruses by infection-transfection methods including recombination site targeting by CRISPR/Cas9 nucleases. J Virol Methods 213:18–25. doi:10.1016/j.jviromet.2014.11.009 25479355

[50] Bellizzi A, Çakır S, Donadoni M, Sariyer R, Liao S, Liu H, Ruan G-X, Gordon J, Khalili K, Sariyer IK. 2024. Suppression of HSV-1 infection and viral reactivation by CRISPR-Cas9 gene editing in 2D and 3D culture models. Mol Ther Nucleic Acids 35:102282. doi:10.1016/j.omtn.2024.102282 39176174 PMC11339036

[51] Xiao J, Deng J, Zhang Q, Ma P, Lv L, Zhang Y, Li C, Zhang Y. 2020. Targeting human cytomegalovirus IE genes by CRISPR/Cas9 nuclease effectively inhibits viral replication and reactivation. Arch Virol 165:1827–1835. doi:10.1007/s00705-020-04687-3 32507978

[52] Natesan JS, Krishnan S. 2021. Using CRISPR technology to inhibit the replication of human cytomegalovirus by deletion of a gene promoter. J Emerg Invest. doi:10.59720/21-060

[53] Braspenning SE, Lebbink RJ, Depledge DP, Schapendonk CME, Anderson LA, Verjans GMGM, Sadaoka T, Ouwendijk WJD. 2021. Mutagenesis of the varicella-zoster virus genome demonstrates that VLT and VLT-ORF63 proteins are dispensable for lytic infection. Viruses 13:2289. doi:10.3390/v13112289 34835095 PMC8619377

[54] Apinda N, Yao Y, Zhang Y, Reddy VRAP, Chang P, Nair V, Sthitmatee N. 2022. CRISPR/Cas9 editing of duck enteritis virus genome for the construction of a recombinant vaccine vector expressing ompH gene of Pasteurella multocida in two novel insertion sites. Vaccines (Basel) 10:686. doi:10.3390/vaccines10050686 35632442 PMC9147145

[55] Li W, Zhang Y, Moffat K, Nair V, Yao Y. 2022. V5 and GFP tagging of viral gene pp38 of Marek’s disease vaccine strain CVI988 using CRISPR/Cas9 editing. Viruses 14:436. doi:10.3390/v14020436 35216029 PMC8879161

[56] Yu W, Liu J, Liu Y, Forlenza M, Chen H. 2024. Application of CRISPR/Cas9 for rapid genome editing of pseudorabies virus and bovine herpesvirus-1. Viruses 16:311. doi:10.3390/v16020311 38400086 PMC10892916

[57] Fu P-F, Cheng X, Su B-Q, Duan L-F, Wang C-R, Niu X-R, Wang J, Yang G-Y, Chu B-B. 2021. CRISPR/Cas9-based generation of a recombinant double-reporter pseudorabies virus and its characterization in vitro and in vivo. Vet Res 52:95. doi:10.1186/s13567-021-00964-4 34174954 PMC8233574

[58] Zhang J-F, Park J-Y, Kim S-W, Choi Y-R, Cha S-Y, Jang H-K, Wei B, Kang M. 2024. Development of a highly efficient CRISPR/Cas9-mediated herpesvirus of Turkey-based vaccine against novel variant infectious bursal disease virus. Vaccines (Basel) 12:226. doi:10.3390/vaccines12030226 38543860 PMC10974780

[59] Yuen K-S, Chan C-P, Wong N-HM, Ho C-H, Ho T-H, Lei T, Deng W, Tsao SW, Chen H, Kok K-H, Jin D-Y. 2015. CRISPR/Cas9-mediated genome editing of Epstein–Barr virus in human cells. J Gen Virol 96:626–636. doi:10.1099/jgv.0.000012 25502645

[60] BeltCappellino A, Majerciak V, Lobanov A, Lack J, Cam M, Zheng Z-M. 2019. CRISPR/Cas9-mediated knockout and in situ inversion of the ORF57 gene from all copies of the Kaposi’s sarcoma-associated herpesvirus genome in BCBL-1 cells. J Virol 93:e00628-19. doi:10.1128/JVI.00628-19 31413125 PMC6803266

[61] Ramanan V, Shlomai A, Cox DBT, Schwartz RE, Michailidis E, Bhatta A, Scott DA, Zhang F, Rice CM, Bhatia SN. 2015. CRISPR/Cas9 cleavage of viral DNA efficiently suppresses hepatitis B virus. Sci Rep 5:10833. doi:10.1038/srep10833 26035283 PMC4649911

[62] Chou Y-Y, Krupp A, Kaynor C, Gaudin R, Ma M, Cahir-McFarland E, Kirchhausen T. 2016. Inhibition of JCPyV infection mediated by targeted viral genome editing using CRISPR/Cas9. Sci Rep 6:36921. doi:10.1038/srep36921 27841295 PMC5107994

[63] Rocchi A, Liao S, Liu H, Chen C, Çakır S, Bellizzi A, Wollebo HS, Sariyer IK, Khalili K. 2025. CRISPR antiviral inhibits neurotrophic JC polyomavirus in 2D and 3D culture models through dual-gRNA excision by SaCas9. Mol Ther Nucleic Acids 36:102556. doi:10.1016/j.omtn.2025.102556 40510594 PMC12159223

[64] Yin C, Zhang T, Qu X, Zhang Y, Putatunda R, Xiao X, Li F, Xiao W, Zhao H, Dai S, Qin X, Mo X, Young W-B, Khalili K, Hu W. 2017. In vivo excision of HIV-1 provirus by saCas9 and multiplex single-guide RNAs in animal models. Mol Ther 25:1168–1186. doi:10.1016/j.ymthe.2017.03.012 28366764 PMC5417847

[65] Xiong J, Tan S, Yu L, Shen H, Qu S, Zhang C, Ren C, Zhu D, Wang H. 2021. E7-targeted nanotherapeutics for key HPV afflicted cervical lesions by employing CRISPR/Cas9 and poly (Beta-Amino Ester). Int J Nanomedicine 16:7609–7622. doi:10.2147/IJN.S335277 34819726 PMC8606985

[66] Noroozi Z, Shamsara M, Valipour E, Esfandyari S, Ehghaghi A, Monfaredan A, Azizi Z, Motevaseli E, Modarressi MH. 2022. Antiproliferative effects of AAV-delivered CRISPR/Cas9-based degradation of the HPV18-E6 gene in HeLa cells. Sci Rep 12:2224. doi:10.1038/s41598-022-06025-w 35140292 PMC8828776

[67] Boutin L, Mosca E, Iseni F. 2022. Efficient method for generating point mutations in the vaccinia virus genome using CRISPR/Cas9. Viruses 14:1559. doi:10.3390/v14071559 35891539 PMC9321979

[68] Kim TH, Shin K, Kim ET. 2025. CRISPR/Cas9-mediated point mutation in essential HSV-1 genes using an IRES-based strategy. J Bacteriol Virol 55:258–269. doi:10.4167/jbv.2025.55.3.258

[69] Shin K, Kim ET. 2025. Efficient CRISPR-based genome editing for inducible degron systems to enable temporal control of protein function in large double-stranded DNA virus genomes. J Microbiol 63:e2504008. doi:10.71150/jm.2504008 41025245 PMC13577094

[70] Jinek M, Chylinski K, Fonfara I, Hauer M, Doudna JA, Charpentier E. 2012. A programmable dual-RNA-guided DNA endonuclease in adaptive bacterial immunity. Science 337:816–821. doi:10.1126/science.1225829 22745249 PMC6286148

[71] Nishimasu H, Shi X, Ishiguro S, Gao L, Hirano S, Okazaki S, Noda T, Abudayyeh OO, Gootenberg JS, Mori H, Oura S, Holmes B, Tanaka M, Seki M, Hirano H, Aburatani H, Ishitani R, Ikawa M, Yachie N, Zhang F, Nureki O. 2018. Engineered CRISPR-Cas9 nuclease with expanded targeting space. Science 361:1259–1262. doi:10.1126/science.aas9129 30166441 PMC6368452

[72] Kleinstiver BP, Pattanayak V, Prew MS, Tsai SQ, Nguyen NT, Zheng Z, Joung JK. 2016. High-fidelity CRISPR-Cas9 nucleases with no detectable genome-wide off-target effects. Nature 529:490–495. doi:10.1038/nature16526 26735016 PMC4851738

[73] Slaymaker IM, Gao L, Zetsche B, Scott DA, Yan WX, Zhang F. 2016. Rationally engineered Cas9 nucleases with improved specificity. Science 351:84–88. doi:10.1126/science.aad5227 26628643 PMC4714946

[74] Xie H, Tang L, He X, Liu X, Zhou C, Liu J, Ge X, Li J, Liu C, Zhao J, Qu J, Song Z, Gu F. 2018. SaCas9 requires 5′‐NNGRRT‐3′ PAM for sufficient cleavage and possesses higher cleavage activity than SpCas9 or FnCpf1 in human cells. Biotechnol J 13:1700561. doi:10.1002/biot.201700561 29247600

[75] Schubert MS, Thommandru B, Woodley J, Turk R, Yan S, Kurgan G, McNeill MS, Rettig GR. 2021. Optimized design parameters for CRISPR Cas9 and Cas12a homology-directed repair. Sci Rep 11:19482. doi:10.1038/s41598-021-98965-y 34593942 PMC8484621

[76] Sharrar A, Arake de Tacca L, Meacham Z, Staples-Ager J, Collingwood T, Rabuka D, Schelle M. 2024. Discovery and engineering of AiEvo2, a novel Cas12a nuclease for human gene editing applications. J Biol Chem 300:105685. doi:10.1016/j.jbc.2024.105685 38272227 PMC10877636

[77] Harrington LB, Burstein D, Chen JS, Paez-Espino D, Ma E, Witte IP, Cofsky JC, Kyrpides NC, Banfield JF, Doudna JA. 2018. Programmed DNA destruction by miniature CRISPR-Cas14 enzymes. Science 362:839–842. doi:10.1126/science.aav4294 30337455 PMC6659742

[78] Labun K, Montague TG, Krause M, Torres Cleuren YN, Tjeldnes H, Valen E. 2019. CHOPCHOP v3: expanding the CRISPR web toolbox beyond genome editing. Nucleic Acids Res 47:W171–W174. doi:10.1093/nar/gkz365 31106371 PMC6602426

[79] Haeussler M, Schönig K, Eckert H, Eschstruth A, Mianné J, Renaud J-B, Schneider-Maunoury S, Shkumatava A, Teboul L, Kent J, Joly J-S, Concordet J-P. 2016. Evaluation of off-target and on-target scoring algorithms and integration into the guide RNA selection tool CRISPOR. Genome Biol 17:148. doi:10.1186/s13059-016-1012-2 27380939 PMC4934014

[80] Moreno-Mateos MA, Vejnar CE, Beaudoin J-D, Fernandez JP, Mis EK, Khokha MK, Giraldez AJ. 2015. CRISPRscan: designing highly efficient sgRNAs for CRISPR-Cas9 targeting in vivo. Nat Methods 12:982–988. doi:10.1038/nmeth.3543 26322839 PMC4589495

[81] Kim HK, Kim Y, Lee S, Min S, Bae JY, Choi JW, Park J, Jung D, Yoon S, Kim HH. 2019. SpCas9 activity prediction by DeepSpCas9, a deep learning-based model with high generalization performance. Sci Adv 5:eaax9249. doi:10.1126/sciadv.aax9249 31723604 PMC6834390

[82] Aoki K, Yamasaki M, Umezono R, Hamamoto T, Kamachi Y. 2024. Systematic comparison of computational tools for sanger sequencing-based genome editing analysis. Cells 13:261. doi:10.3390/cells13030261 38334653 PMC10854981

[83] Konstantakos V, Nentidis A, Krithara A, Paliouras G. 2022. CRISPR-Cas9 gRNA efficiency prediction: an overview of predictive tools and the role of deep learning. Nucleic Acids Res 50:3616–3637. doi:10.1093/nar/gkac192 35349718 PMC9023298

[84] Lee CM, Davis TH, Bao G. 2018. Examination of CRISPR/Cas9 design tools and the effect of target site accessibility on Cas9 activity. Exp Physiol 103:456–460. doi:10.1113/EP086043 28303677 PMC7266697

[85] Ran FA, Hsu PD, Wright J, Agarwala V, Scott DA, Zhang F. 2013. Genome engineering using the CRISPR-Cas9 system. Nat Protoc 8:2281–2308. doi:10.1038/nprot.2013.143 24157548 PMC3969860

[86] Tsai SQ, Zheng Z, Nguyen NT, Liebers M, Topkar VV, Thapar V, Wyvekens N, Khayter C, Iafrate AJ, Le LP, Aryee MJ, Joung JK. 2015. GUIDE-seq enables genome-wide profiling of off-target cleavage by CRISPR-Cas nucleases. Nat Biotechnol 33:187–197. doi:10.1038/nbt.3117 25513782 PMC4320685

[87] Paquet D, Kwart D, Chen A, Sproul A, Jacob S, Teo S, Olsen KM, Gregg A, Noggle S, Tessier-Lavigne M. 2016. Efficient introduction of specific homozygous and heterozygous mutations using CRISPR/Cas9. Nature 533:125–129. doi:10.1038/nature17664 27120160

[88] Wu X, Kriz AJ, Sharp PA. 2014. Target specificity of the CRISPR-Cas9 system. Quant Biol 2:59–70. doi:10.1007/s40484-014-0030-x 25722925 PMC4338555

[89] Liu X, Homma A, Sayadi J, Yang S, Ohashi J, Takumi T. 2016. Sequence features associated with the cleavage efficiency of CRISPR/Cas9 system. Sci Rep 6:19675. doi:10.1038/srep19675 26813419 PMC4728555

[90] Tang Y-D, Guo J-C, Wang T-Y, Zhao K, Liu J-T, Gao J-C, Tian Z-J, An T-Q, Cai X-H. 2018. CRISPR/Cas9-mediated 2-sgRNA cleavage facilitates pseudorabies virus editing. FASEB J 32:4293–4301. doi:10.1096/fj.201701129R 29509513

[91] Quadros RM, Miura H, Harms DW, Akatsuka H, Sato T, Aida T, Redder R, Richardson GP, Inagaki Y, Sakai D, Buckley SM, Seshacharyulu P, Batra SK, Behlke MA, Zeiner SA, Jacobi AM, Izu Y, Thoreson WB, Urness LD, Mansour SL, Ohtsuka M, Gurumurthy CB. 2017. Easi-CRISPR: a robust method for one-step generation of mice carrying conditional and insertion alleles using long ssDNA donors and CRISPR ribonucleoproteins. Genome Biol 18:92. doi:10.1186/s13059-017-1220-4 28511701 PMC5434640

[92] Jin Y-Y, Zhang P, Liu L-L, Zhao X, Hu X-Q, Liu S-Z, Li Z-K, Liu Q, Wang J-Q, Hao D-L, Zhang Z-Q, Chen H-Z, Liu D-P. 2024. Enhancing homology-directed repair efficiency with HDR-boosting modular ssDNA donor. Nat Commun 15:6843. doi:10.1038/s41467-024-50788-x 39122671 PMC11315919

[93] Miura H, Quadros RM, Gurumurthy CB, Ohtsuka M. 2018. Easi-CRISPR for creating knock-in and conditional knockout mouse models using long ssDNA donors. Nat Protoc 13:195–215. doi:10.1038/nprot.2017.153 29266098 PMC6058056

[94] Dokshin GA, Ghanta KS, Piscopo KM, Mello CC. 2018. Robust genome editing with short single-stranded and long, partially single-stranded DNA donors in Caenorhabditis elegans. Genetics 210:781–787. doi:10.1534/genetics.118.301532 30213854 PMC6218216

[95] Ghanta KS, Mello CC. 2020. Melting dsDNA donor molecules greatly improves precision genome editing in Caenorhabditis elegans. Genetics 216:643–650. doi:10.1534/genetics.120.303564 32963112 PMC7648581

[96] Zhang J-P, Li X-L, Li G-H, Chen W, Arakaki C, Botimer GD, Baylink D, Zhang L, Wen W, Fu Y-W, Xu J, Chun N, Yuan W, Cheng T, Zhang X-B. 2017. Efficient precise knockin with a double cut HDR donor after CRISPR/Cas9-mediated double-stranded DNA cleavage. Genome Biol 18:35. doi:10.1186/s13059-017-1164-8 28219395 PMC5319046

[97] Liao H, Wu J, VanDusen NJ, Li Y, Zheng Y. 2024. CRISPR-Cas9-mediated homology-directed repair for precise gene editing. Mol Ther Nucleic Acids 35:102344. doi:10.1016/j.omtn.2024.102344 39494147 PMC11531618

[98] Nishiyama J, Mikuni T, Yasuda R. 2017. Virus-mediated genome editing via homology-directed repair in mitotic and postmitotic cells in mammalian brain. Neuron 96:755–768. doi:10.1016/j.neuron.2017.10.004 29056297 PMC5691606

[99] Wang Y, Wang Y, Chang T, Huang H, Yee J-K. 2017. Integration-defective lentiviral vector mediates efficient gene editing through homology-directed repair in human embryonic stem cells. Nucleic Acids Res 45:e29–e29. doi:10.1093/nar/gkw1057 27899664 PMC5389720

[100] Lotfi M, Morshedi Rad D, Mashhadi SS, Ashouri A, Mojarrad M, Mozaffari-Jovin S, Farrokhi S, Hashemi M, Lotfi M, Ebrahimi Warkiani M, Abbaszadegan MR. 2023. Recent advances in CRISPR/Cas9 delivery approaches for therapeutic gene editing of stem cells. Stem Cell Rev Rep 19:2576–2596. doi:10.1007/s12015-023-10585-3 37723364 PMC10661828

[101] Kaupbayeva B, Tsoy A, Safarova Yantsen Y, Nurmagambetova A, Murata H, Matyjaszewski K, Askarova S. 2024. Unlocking genome editing: advances and obstacles in CRISPR/Cas delivery technologies. J Funct Biomater 15:324. doi:10.3390/jfb15110324 39590528 PMC11595195

[102] Huang J, Zhou Y, Li J, Lu A, Liang C. 2022. CRISPR/Cas systems: delivery and application in gene therapy. Front Bioeng Biotechnol 10:10. doi:10.3389/fbioe.2022.942325 PMC9723151 36483767

[103] Chavez M, Rane DA, Chen X, Qi LS. 2023. Stable expression of large transgenes via the knock-in of an integrase-deficient lentivirus. Nat Biomed Eng 7:661–671. doi:10.1038/s41551-023-01037-x 37127707

[104] Yu X, Liang X, Xie H, Kumar S, Ravinder N, Potter J, de Mollerat du Jeu X, Chesnut JD. 2016. Improved delivery of Cas9 protein/gRNA complexes using lipofectamine CRISPRMAX. Biotechnol Lett 38:919–929. doi:10.1007/s10529-016-2064-9 26892225 PMC4853464

[105] Kim S, Kim D, Cho SW, Kim J, Kim J-S. 2014. Highly efficient RNA-guided genome editing in human cells via delivery of purified Cas9 ribonucleoproteins. Genome Res 24:1012–1019. doi:10.1101/gr.171322.113 24696461 PMC4032847

[106] Cheng Q, Xia J, Wang K, Zhang Y, Chen Y, Zhong Q, Wang X, Wu Q. 2022. CRISPR/Cas9 ribonucleoprotein (RNP) complex enables higher viability of transfected cells in genome editing of acute myeloid cells. Ann Transl Med 10:862–862. doi:10.21037/atm-22-3279 36111017 PMC9469150

[107] Bamundo M, Palumbo S, D’Auria L, Missero C, Di Girolamo D. 2024. CRISPR/Cas9 ribonucleoprotein nucleofection for genome editing in primary human keratinocytes: knockouts, deletions, and homology-directed repair mutagenesis. Curr Protoc 4:e70056. doi:10.1002/cpz1.70056 39601181 PMC11600394

[108] Roth TL, Puig-Saus C, Yu R, Shifrut E, Carnevale J, Li PJ, Hiatt J, Saco J, Krystofinski P, Li H, et al.. 2018. Reprogramming human T cell function and specificity with non-viral genome targeting. Nature 559:405–409. doi:10.1038/s41586-018-0326-5 29995861 PMC6239417

[109] Rai R, Romito M, Rivers E, Turchiano G, Blattner G, Vetharoy W, Ladon D, Andrieux G, Zhang F, Zinicola M, Leon-Rico D, Santilli G, Thrasher AJ, Cavazza A. 2020. Targeted gene correction of human hematopoietic stem cells for the treatment of Wiskott - Aldrich syndrome. Nat Commun 11. doi:10.1038/s41467-020-17626-2 PMC7423939 32788576

[110] Dong W, Kantor B. 2021. Lentiviral vectors for delivery of gene-editing systems based on CRISPR/Cas: current state and perspectives. Viruses 13:1288. doi:10.3390/v13071288 34372494 PMC8310029

[111] Azhagiri MKK, Babu P, Venkatesan V, Thangavel S. 2021. Homology-directed gene-editing approaches for hematopoietic stem and progenitor cell gene therapy. Stem Cell Res Ther 12. doi:10.1186/s13287-021-02565-6 PMC8428126 34503562

[112] Yang J, Guo F, Chin HS, Chen GB, Zhang Z, Williams L, Kueh AJ, Chow PKH, Herold MJ, Fu NY. 2025. Rapid and robust generation of homozygous fluorescent reporter knock-in cell pools by CRISPR-Cas9. Cells 14:1165. doi:10.3390/cells14151165 40801599 PMC12346671

[113] Moço PD, Aharony N, Kamen A. 2020. Adeno-associated viral vectors for homology-directed generation of CAR-T cells. Biotechnol J 15:e1900286. doi:10.1002/biot.201900286 31642193

[114] Duddy G, Courtis K, Horwood J, Olsen J, Horsler H, Hodgson T, Varsani-Brown S, Abdullah A, Denti L, Lane H, Delaqua F, Janzen J, Strom M, Rosewell I, Crawley K, Davies B. 2024. Donor template delivery by recombinant adeno-associated virus for the production of knock-in mice. BMC Biol 22. doi:10.1186/s12915-024-01834-z PMC10836013 38302906

[115] Fu Y-W, Dai X-Y, Wang W-T, Yang Z-X, Zhao J-J, Zhang J-P, Wen W, Zhang F, Oberg KC, Zhang L, Cheng T, Zhang X-B. 2021. Dynamics and competition of CRISPR–Cas9 ribonucleoproteins and AAV donor-mediated NHEJ, MMEJ and HDR editing. Nucleic Acids Res 49:969–985. doi:10.1093/nar/gkaa1251 33398341 PMC7826255

[116] Hanlon KS, Kleinstiver BP, Garcia SP, Zaborowski MP, Volak A, Spirig SE, Muller A, Sousa AA, Tsai SQ, Bengtsson NE, Lööv C, Ingelsson M, Chamberlain JS, Corey DP, Aryee MJ, Joung JK, Breakefield XO, Maguire CA, György B. 2019. High levels of AAV vector integration into CRISPR-induced DNA breaks. Nat Commun 10. doi:10.1038/s41467-019-12449-2 PMC6769011 31570731

[117] Suoranta T, Laham-Karam N, Ylä-Herttuala S. 2022. Strategies to improve safety profile of AAV vectors. Front Mol Med 2:1054069. doi:10.3389/fmmed.2022.1054069 39086961 PMC11285686

[118] Ling C, Yu C, Wang C, Yang M, Yang H, Yang K, He Y, Shen Y, Tang S, Yu X, Zhou Z, Zhou S, Zhou J, Zhu L, Li J. 2024. rAAV capsid mutants eliminate leaky expression from DNA donor template for homologous recombination. Nucleic Acids Res 52:6518–6531. doi:10.1093/nar/gkae401 38783157 PMC11194064

[119] Gwiazda KS, Grier AE, Sahni J, Burleigh SM, Martin U, Yang JG, Popp NA, Krutein MC, Khan IF, Jacoby K, Jensen MC, Rawlings DJ, Scharenberg AM. 2016. High efficiency CRISPR/Cas9-mediated gene editing in primary human T-cells using mutant adenoviral E4orf6/E1b55k “helper” proteins. Mol Ther 24:1570–1580. doi:10.1038/mt.2016.105 27203437 PMC5113096

[120] Tan E, Chin CSH, Lim ZFS, Ng SK. 2021. HEK293 cell line as a platform to produce recombinant proteins and viral vectors. Front Bioeng Biotechnol 9:9. doi:10.3389/fbioe.2021.796991 PMC8711270 34966729

[121] Kingston RE, Chen CA, Okayama H. 1999. Calcium phosphate transfection. CP in Immunology 31. doi:10.1002/0471142735.im1013s31 18432676

[122] Gillmore JD. 2021. CRISPR-Cas9 in vivo gene editing for transthyretin amyloidosis. N Engl J Med 385:1721–1723. doi:10.1056/NEJMc2114592 34215024

[123] Dubey AK, Mostafavi E. 2023. Biomaterials-mediated CRISPR/Cas9 delivery: recent challenges and opportunities in gene therapy. Front Chem 11:11. doi:10.3389/fchem.2023.1259435 PMC10568484 37841202

[124] Öktem M, Mastrobattista E, de Jong OG. 2023 Amphipathic cell-penetrating peptide-aided delivery of Cas9 RNP for in vitro gene editing and correction. Pharmaceutics 15:2500. doi:10.3390/pharmaceutics15102500 37896260 PMC10609989

[125] de Morais CCP de L, Correia EM, Bonamino MH, Vasconcelos ZFM de. 2024. Cell-penetrating peptides and CRISPR-Cas9: a combined strategy for human genetic disease therapy. Hum Gene Ther 35:781–797. doi:10.1089/hum.2024.020 39276086 PMC11511780

[126] Hryhorowicz M, Grześkowiak B, Mazurkiewicz N, Śledziński P, Lipiński D, Słomski R. 2019. Improved delivery of CRISPR/Cas9 system using magnetic nanoparticles into porcine fibroblast. Mol Biotechnol 61:173–180. doi:10.1007/s12033-018-0145-9 30560399

[127] Rohiwal SS, Dvorakova N, Klima J, Vaskovicova M, Senigl F, Slouf M, Pavlova E, Stepanek P, Babuka D, Benes H, Ellederova Z, Stieger K. 2020. Polyethylenimine based magnetic nanoparticles mediated non-viral CRISPR/Cas9 system for genome editing. Sci Rep 10:4619. doi:10.1038/s41598-020-61465-6 32165679 PMC7067791

[128] Cho H-Y, Yoo M, Pongkulapa T, Rabie H, Muotri AR, Yin PT, Choi J-W, Lee K-B. 2024. Magnetic nanoparticle-assisted non-viral CRISPR-Cas9 for enhanced genome editing to treat rett syndrome. Adv Sci (Weinh) 11:e2306432. doi:10.1002/advs.202306432 38647391 PMC11200027

[129] Wu Y, Battalapalli D, Hakeem MJ, Selamneni V, Zhang P, Draz MS, Ruan Z. 2021. Engineered CRISPR-Cas systems for the detection and control of antibiotic-resistant infections. J Nanobiotechnology 19:401. doi:10.1186/s12951-021-01132-8 34863214 PMC8642896

[130] Hustedt N, Durocher D. 2017. The control of DNA repair by the cell cycle. Nat Cell Biol 19:1–9. doi:10.1038/ncb3452 28008184

[131] Lee ABC, Tan M-H, Chai CLL. 2022. Small-molecule enhancers of CRISPR-induced homology-directed repair in gene therapy: a medicinal chemist’s perspective. Drug Discov Today 27:2510–2525. doi:10.1016/j.drudis.2022.06.006 35738528

[132] Li G, Wang H, Zhang X, Wu Z, Yang H. 2021. A Cas9–transcription factor fusion protein enhances homology-directed repair efficiency. J Biol Chem 296:100525. doi:10.1016/j.jbc.2021.100525 33689695 PMC8042446

[133] Lin S, Staahl BT, Alla RK, Doudna JA. 2014. Enhanced homology-directed human genome engineering by controlled timing of CRISPR/Cas9 delivery. eLife 3:e04766. doi:10.7554/eLife.04766 25497837 PMC4383097

[134] Wienert B, Nguyen DN, Guenther A, Feng SJ, Locke MN, Wyman SK, Shin J, Kazane KR, Gregory GL, Carter MAM, Wright F, Conklin BR, Marson A, Richardson CD, Corn JE. 2020. Timed inhibition of CDC7 increases CRISPR-Cas9 mediated templated repair. Nat Commun 11:2109. doi:10.1038/s41467-020-15845-1 32355159 PMC7193628

[135] Davis AJ, Chen DJ. 2013. DNA double strand break repair via non-homologous end-joining. Transl Cancer Res 2:130–143. doi:10.3978/j.issn.2218-676X.2013.04.02 24000320 PMC3758668

[136] Loparo JJ. 2023. Holding it together: DNA end synapsis during non-homologous end joining. DNA Repair (Amst) 130:103553. doi:10.1016/j.dnarep.2023.103553 37572577 PMC10530278

[137] Riesenberg S, Maricic T. 2018. Targeting repair pathways with small molecules increases precise genome editing in pluripotent stem cells. Nat Commun 9:2164. doi:10.1038/s41467-018-04609-7 29867139 PMC5986859

[138] Bischoff N, Wimberger S, Maresca M, Brakebusch C. 2020. Improving precise CRISPR genome editing by small molecules: is there a magic potion? Cells 9:1318. doi:10.3390/cells9051318 32466303 PMC7291049

[139] Yao X, Wang X, Hu X, Liu Z, Liu J, Zhou H, Shen X, Wei Y, Huang Z, Ying W, Wang Y, Nie Y-H, Zhang C-C, Li S, Cheng L, Wang Q, Wu Y, Huang P, Sun Q, Shi L, Yang H. 2017. Homology-mediated end joining-based targeted integration using CRISPR/Cas9. Cell Res 27:801–814. doi:10.1038/cr.2017.76 28524166 PMC5518881

[140] Wimberger S, Akrap N, Firth M, Brengdahl J, Engberg S, Schwinn MK, Slater MR, Lundin A, Hsieh P-P, Li S, et al.. 2023 Simultaneous inhibition of DNA-PK and Polϴ improves integration efficiency and precision of genome editing. Nat Commun 14. doi:10.1038/s41467-023-40344-4 PMC10425386 37580318

[141] Fok JHL, Ramos-Montoya A, Vazquez-Chantada M, Wijnhoven PWG, Follia V, James N, Farrington PM, Karmokar A, Willis SE, Cairns J, Nikkilä J, Beattie D, Lamont GM, Finlay MRV, Wilson J, Smith A, O’Connor LO, Ling S, Fawell SE, O’Connor MJ, Hollingsworth SJ, Dean E, Goldberg FW, Davies BR, Cadogan EB. 2019. AZD7648 is a potent and selective DNA-PK inhibitor that enhances radiation, chemotherapy and olaparib activity. Nat Commun 10:5065. doi:10.1038/s41467-019-12836-9 31699977 PMC6838110

[142] Selvaraj S, Feist WN, Viel S, Vaidyanathan S, Dudek AM, Gastou M, Rockwood SJ, Ekman FK, Oseghale AR, Xu L, Pavel-Dinu M, Luna SE, Cromer MK, Sayana R, Gomez-Ospina N, Porteus MH. 2024. High-efficiency transgene integration by homology-directed repair in human primary cells using DNA-PKcs inhibition. Nat Biotechnol 42:731–744. doi:10.1038/s41587-023-01888-4 37537500

[143] Cullot G, Aird EJ, Schlapansky MF, Yeh CD, van de Venn L, Vykhlyantseva I, Kreutzer S, Mailänder D, Lewków B, Klermund J, Montellese C, Biserni M, Aeschimann F, Vonarburg C, Gehart H, Cathomen T, Corn JE. 2025. Genome editing with the HDR-enhancing DNA-PKcs inhibitor AZD7648 causes large-scale genomic alterations. Nat Biotechnol 43:1778–1782. doi:10.1038/s41587-024-02488-6 39604565 PMC12611759

[144] Maruyama T, Dougan SK, Truttmann MC, Bilate AM, Ingram JR, Ploegh HL. 2015. Increasing the efficiency of precise genome editing with CRISPR-Cas9 by inhibition of nonhomologous end joining. Nat Biotechnol 33:538–542. doi:10.1038/nbt.3190 25798939 PMC4618510

[145] Shams F, Bayat H, Mohammadian O, Mahboudi S, Vahidnezhad H, Soosanabadi M, Rahimpour A. 2022. Advance trends in targeting homology-directed repair for accurate gene editing: an inclusive review of small molecules and modified CRISPR-Cas9 systems. Bioimpacts 12:371–391. doi:10.34172/bi.2022.23871 35975201 PMC9376165

[146] Anuchina AA, Zaynitdinova MI, Demchenko AG, Evtushenko NA, Lavrov AV, Smirnikhina SA. 2023. Bridging gaps in HDR Improvement: the role of MAD2L2, SCAI, and SCR7. Int J Mol Sci 24:6704. doi:10.3390/ijms24076704 37047677 PMC10095018

[147] Jayathilaka K, Sheridan SD, Bold TD, Bochenska K, Logan HL, Weichselbaum RR, Bishop DK, Connell PP. 2008. A chemical compound that stimulates the human homologous recombination protein RAD51. Proc Natl Acad Sci USA 105:15848–15853. doi:10.1073/pnas.0808046105 18840682 PMC2572930

[148] Song J, Yang D, Xu J, Zhu T, Chen YE, Zhang J. 2016. RS-1 enhances CRISPR/Cas9- and TALEN-mediated knock-in efficiency. Nat Commun 7:10548. doi:10.1038/ncomms10548 26817820 PMC4738357

[149] Liu N, Zhou L, Lin G, Hu Y, Jiao Y, Wang Y, Liu J, Yang S, Yao S. 2022. HDAC inhibitors improve CRISPR-Cas9 mediated prime editing and base editing. Mol Ther Nucleic Acids 29:36–46. doi:10.1016/j.omtn.2022.05.036 35784015 PMC9207553

[150] Ma X, Chen X, Jin Y, Ge W, Wang W, Kong L, Ji J, Guo X, Huang J, Feng X-H, Fu J, Zhu S. 2018. Small molecules promote CRISPR-Cpf1-mediated genome editing in human pluripotent stem cells. Nat Commun 9. doi:10.1038/s41467-018-03760-5 PMC5880812 29610531

[151] Chan HY, Xing X, Kraus P, Yap SP, Ng P, Lim SL, Lufkin T. 2011. Comparison of IRES and F2A-based locus-specific multicistronic expression in stable mouse lines. PLoS One 6:e28885. doi:10.1371/journal.pone.0028885 22216134 PMC3244433

[152] Komar AA, Hatzoglou M. 2011. Cellular IRES-mediated translation. Cell Cycle 10:229–240. doi:10.4161/cc.10.2.14472 21220943 PMC3048795

[153] Liu Z, Chen O, Wall JBJ, Zheng M, Zhou Y, Wang L, Ruth Vaseghi H, Qian L, Liu J. 2017. Systematic comparison of 2A peptides for cloning multi-genes in a polycistronic vector. Sci Rep 7. doi:10.1038/s41598-017-02460-2 PMC5438344 28526819

[154] Kim JH, Lee S-R, Li L-H, Park H-J, Park J-H, Lee KY, Kim M-K, Shin BA, Choi S-Y. 2011. High cleavage efficiency of a 2A peptide derived from porcine teschovirus-1 in human cell lines, zebrafish and mice. PLoS One 6:e18556. doi:10.1371/journal.pone.0018556 21602908 PMC3084703

[155] Wang S, Li Y, Zhong L, Wu K, Zhang R, Kang T, Wu S, Wu Y. 2022. Efficient gene editing through an intronic selection marker in cells. Cell Mol Life Sci 79:111. doi:10.1007/s00018-022-04152-1 35098362 PMC8801403

[156] Reuven N, Shaul Y. 2022. Selecting for CRISPR-edited knock-in cells. IJMS 23:11919. doi:10.3390/ijms231911919 36233222 PMC9569436

[157] Sharma I, Hall K, Moonah S. 2025. CRISPR genome editing using a combined positive and negative selection system. PLoS One 20:e0321881. doi:10.1371/journal.pone.0321881 40327602 PMC12054870

[158] Grosche L, Döhner K, Düthorn A, Hickford-Martinez A, Steinkasserer A, Sodeik B. 2019. Herpes simplex virus type 1 propagation, titration and single-step growth curves. Bio Protoc 9. doi:10.21769/BioProtoc.3441 PMC7853997 33654936

[159] Nguyen H-M, Sah N, Humphrey MRM, Rabkin SD, Saha D. 2021. Growth, purification, and titration of oncolytic herpes simplex virus. J Vis Exp 171. doi:10.3791/62677 PMC8447238 34057449

[160] Wang H-M, Xu S-J, Cai B-Y, Qiu W-Y, Lu H, Tang Y-D. 2025. Highly efficient gene editing of Feline herpesvirus 1 using CRISPR/Cas9 combined with FACS. Front Cell Infect Microbiol 15:1660446. doi:10.3389/fcimb.2025.1660446 40904932 PMC12401898

[161] Ramakrishnan MA. 2016. Determination of 50% endpoint titer using a simple formula. WJV 5:85. doi:10.5501/wjv.v5.i2.85 27175354 PMC4861875

[162] Lei C, Yang J, Hu J, Sun X. 2021. On the calculation of TCID50 for quantitation of virus infectivity. Virol Sin 36:141–144. doi:10.1007/s12250-020-00230-5 32458296 PMC7973348

[163] Loveday EK, Sanchez HS, Thomas MM, Chang CB. 2022. Single-cell infection of influenza A virus using drop-based microfluidics. Microbiol Spectr 10:e0099322. doi:10.1128/spectrum.00993-22 36125315 PMC9603537

[164] Chaipan C, Pryszlak A, Dean H, Poignard P, Benes V, Griffiths AD, Merten CA. 2017. Single-virus droplet microfluidics for high-throughput screening of neutralizing epitopes on HIV particles. Cell Chem Biol 24:751–757. doi:10.1016/j.chembiol.2017.05.009 28552581

[165] Simpson BP, Yrigollen CM, Izda A, Davidson BL. 2023. Targeted long-read sequencing captures CRISPR editing and AAV integration outcomes in brain. Mol Ther 31:760–773. doi:10.1016/j.ymthe.2023.01.004 36617193 PMC10014281

